# Characterization of *Mycobacterium smegmatis sigF* mutant and its regulon: overexpression of SigF antagonist (MSMEG_1803) in *M. smegmatis* mimics *sigF* mutant phenotype, loss of pigmentation, and sensitivity to oxidative stress

**DOI:** 10.1002/mbo3.288

**Published:** 2015-10-05

**Authors:** Anirudh K. Singh, Debashis Dutta, Vandana Singh, Vishal Srivastava, Rajesh K. Biswas, Bhupendra N. Singh

**Affiliations:** ^1^ Division of MicrobiologyCSIR‐Central Drug Research InstituteLucknow226031India; ^2^ Centre for Microbial PathogenesisResearch Institute at Nationwide Children's Hospital700 Children's DriveColumbusOhio43205; ^3^ The Genomics InstituteWadsworth CenterNew York State Department of Health 150Scottland Ave, AlbanyNew York12208

**Keywords:** Anti‐SigF, anti‐SigF antagonists, *M. smegmatis*, oxidative stress, SigF regulon

## Abstract

In *Mycobacterium smegmatis*,* sigF* is widely expressed during different growth stages and plays role in adaptation to stationary phase and oxidative stress. Using a *sigF* deletion mutant of *M. smegmatis* mc^2^155, we demonstrate that SigF is not essential for growth of bacterium. Deletion of *sigF* results in loss of carotenoid pigmentation which rendered increased susceptibility to H_2_O_2_ induced oxidative stress in *M. smegmatis*. SigF modulates the cell surface architecture and lipid biosynthesis extending the repertoire of SigF function in this species. *M. smegmatis* SigF regulon included variety of genes expressed during exponential and stationary phases of growth and those responsible for oxidative stress, lipid biosynthesis, energy, and central intermediary metabolism. Furthermore, we report the identification of a SigF antagonist, an anti‐sigma factor (RsbW), which upon overexpression in *M. smegmatis* wild type strain produced a phenotype similar to *M. smegmatis* mc^2^155 Δ*sigF* strain. The SigF‐anti‐SigF interaction is duly validated using bacterial two‐hybrid and pull down assays. In addition, anti‐sigma factor antagonists, RsfA and RsfB were identified and their interactions with anti‐sigma factor were experimentally validated. Identification of these proteins will help decode regulatory circuit of this alternate sigma factor.

## Introduction

*Mycobacterium smegmatis*, a fast‐growing saprophytic environmental bacterium, is used as a surrogate to study mycobacterial physiology and gene regulation as it is easy to culture in laboratory conditions. Owing to its habitat, *M. smegmatis* encounters more diverse conditions than its pathogenic counterparts and consequently its genome (6.98 Mb) has expanded nearly twice to the size of *M*. *tuberculosis* (4.4 Mb) to accommodate more genes. There is an unusual expansion of several genes which have acquired many paralogs unlike in other mycobacterial species (Waagmeester et al. 2005). There are 28 sigma factor genes in *M. smegmatis* in contrast with 13 reported in *M. tuberculosis* (Cole et al. 1998; Waagmeester et al. 2005; Rodrigue et al. 2006) and there are seven paralogs of sigma factor *sigH*, which are differentially expressed in *M. smegmatis* (Waagmeester et al. 2005; Singh and Singh 2009). Sigma factors reversibly associate with RNA polymerase and allow them to specifically direct the expression of specific set of genes. *M. smegmatis* genome encodes one of each group I, II, and III sigma factors represented by SigA, SigB, and SigF, respectively, and 25 of group IV sigma factors (Kapopoulou et al. 2011). SigA, the primary sigma factor in both *M. smegmatis* and *M. tuberculosis*, is essential for bacterial viability (Gomez et al. 1998), while SigB, the primary‐like sigma factor is very similar to SigA and is dispensable for growth in *M. smegmatis* (Fontán et al. 2009). SigF (group III) and extracytoplasmic function (ECF) sigma factors (group IV) constitute alternate sigma factors which enable adaptation to a range of external and internal stimuli. Locus for *sigA*,* sigB*,* sigD*,* sigE*,* sigF*,* sigG,* and *sigH* are well conserved in *M. smegmatis* and *M. tuberculosis* (Sachdeva et al. 2010).

Earlier, the *sigF* was reported as a late‐stage specific sigma factor, present only in the genomes of slow‐growing pathogenic mycobacteria (DeMaio et al. 1996, 1997). *M. tuberculosis sigF* was found strongly induced within cultured human macrophages, during stationary phase of growth, upon exposure to cold shock, nutrient starvation, and several antibiotics (Graham and Clark‐Curtiss 1999; Michele et al. 1999; Betts et al. 2002). *M. tuberculosis* Δ*sigF* strain grew to a threefold higher density in stationary phase than the wild‐type strain (Chen et al. 2000), but showed almost similar sensitivity to heat shock, cold shock, and hypoxia relative to the parental strain (Geiman et al. 2004; Hartkoorn et al. 2010). *M. tuberculosis* Δ*sigF* strain was attenuated for virulence in a mouse infection model despite persistence at high bacterial load in lungs compared with the isogenic wild type (Geiman et al. 2004). Overexpression of *sigF* in *M. tuberculosis* resulted in the differential regulation of many cell wall‐associated proteins and other genes involved in the biosynthesis and degradation of surface polysaccharides and lippolysaccharides, believed to play important roles in host‐pathogen interactions (Williams et al. 2007; Hartkoorn et al. 2010). However, we earlier demonstrated that, *sigF* is conserved in all the mycobacterial species analyzed and proposed that apart from regulating the expression of virulence genes in pathogenic mycobacteria, SigF is likely to play more roles in mycobacterial physiology (Singh and Singh 2008).

In *M. smegmatis*,* sigF* is widely expressed during different growth stages (Singh and Singh 2008). *M. smegmatis sigF* is transcriptionally induced in response to nutrient depletion, cold shock and upon exposure to agents that damage cell wall architecture, like SDS and antibiotics, isoniazid, and ethambutol (Singh and Singh 2008; Gebhard et al. 2008). A *sigF* mutant of *M. smegmatis* ATCC 607 strain showed higher transformation efficiency, lack of carotenoid pigmentation, and increased susceptibility to hydrogen peroxide mediated oxidative stress (Provvedi et al. 2008). SigF in *M. smegmatis* plays role in adaptation to stationary phase, heat, and oxidative stress (Hümpel et al. 2010). While both these studies demonstrate the role of *M*. *smegmatis* SigF in oxidative stress, molecular basis of this increased sensitivity to hydrogen peroxide remains unclear. Furthermore, proteins involved in post‐translation regulation of *M. smegmatis* SigF activity are not characterized, making it difficult to define the regulation circuitry of this alternate sigma factor. Using an insertion deletion mutant of *M. smegmatis* mc^2^ 155 *sigF*, we demonstrate that SigF in *M. smegmatis* modulates the cell surface architecture and lipid biosynthesis, extending the repertoire of SigF function in this species. We also demonstrate that the increased sensitivity of the *sigF* mutant to H_2_O_2_ mediated oxidative stress is primarily due to loss of the carotenoid pigment. Furthermore, we report the identification of a SigF antagonist, an anti‐sigma factor (RsbW), which upon overexpression in *M. smegmatis* wild type strain produced a phenotype similar to *M. smegmatis* mc^2^155 Δ*sigF* strain. The SigF‐anti‐SigF interaction was duly confirmed using bacterial two‐hybrid system and pull down assay. In addition, anti‐sigma factor antagonists, RsfA and RsfB were identified and their interactions with anti‐sigma factor were verified using two‐hybrid system.

## Results and Discussion

### Construction of *Mycobacterium smegmatis sigF* knockout mutant and its complementation

The *sigF* deletion (Δ*sigF*) mutant was created by replacing *sigF* ORF with the hygromycin (*hyg*) resistance cassette and molecularly validated (see supplemental material, Fig. S1) as detailed in methods. One of the Δ*sigF* mutants referred as SFKO1 has been studied and described throughout this manuscript. The SFKO1 was complemented with the *sigF* gene, cloned downstream of *hsp60* promoter, at an ectopic locus in the SFKO1 genome. The *sigF* complemented strain is designated as SFKO1/*sigF*.

### Role of SigF in stress responses

The effect of *sigF* deletion on in vitro growth was monitored by comparing the growth of the SFKO1 strain to the wild type *M. smegmatis*. Both strains were allowed to grow in different media for extended length of time; the *sigF* mutant strain grew slightly faster than the wild type, attained higher cell density with reduced lag phase, but displayed similar growth characteristics afterwards till extended stationary phase of growth (data not shown). This suggests that the *sigF* is dispensable for the growth of *M. smegmatis* under normal physiological conditions. These results are in line with the earlier findings (Provvedi et al. 2008).

SigF has been described as a stress‐response sigma factor in slow‐growing mycobacteria (DeMaio et al. 1996). Previously, we had shown that *sigF* is transcriptionally induced in *M*. *smegmatis* in response to cold shock, nutrient starvation and after treatment with SDS and antimycobacterial drugs like isoniazid and ethambutol (Singh and Singh 2008). We examined whether SigF is required for survival of *M. smegmatis* during these stress conditions. No significant differences in survival were noticed between the *sigF* mutant and the wild type strain under these stress conditions (data not shown). Gebhard et al. (Gebhard et al. 2008) had reported that SigF is required for survival against heat shock and acidic stress in *M. smegmatis*. We did not test the acidic stress but upon heat shock no appreciable difference in survival of *sigF* mutant was noticed in comparison to the wild type strain. We checked the *sigF* deletion mutants of both *M. smegmatis* mc^2^155 (SFKO1) and *M. smegmatis* ATCC 607 strains. One of the reasons of this difference could be the temperature as we tested the survival, based on our earlier studies (Singh and Singh 2008, 2009), at 45°C while they used 50°C for heat stress in their studies.

**Figure 1:**
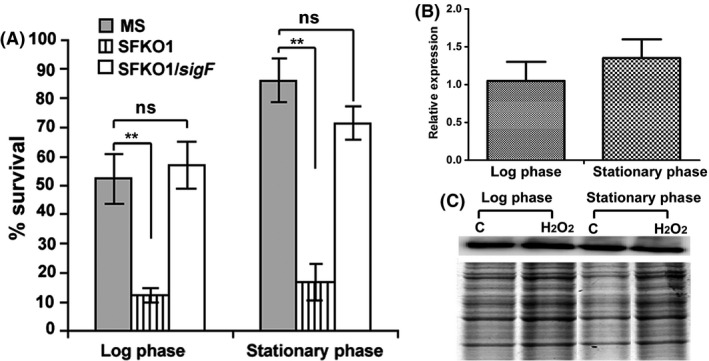
Oxidative stress susceptibility of Δ*sigF* mutant despite similar level of *sigF* expression at RNA and protein level during oxidative stress. (A) *Mycobacterium smegmatis* WT (MS), MSΔ*sigF* mutant (SFKO1) and MSΔ*sigF/sigF* complemented (SFKO1/*sigF*) strains were subjected to oxidative stress (10 mmol L^−1^ H_2_O_2_) and their percent survival were calculated as described in methods. Susceptibility of Δ*sigF* mutant to oxidative stress is completely restored after complementation with *sigF*. Data were collected from three different experiments. The mean values and standard deviations were plotted for each set of data. **P <* *0.01 relative to *M. smegmatis* wild type (MS) as determined by paired *t*‐test. (B) Quantitative real time RT‐PCR analysis of *sigF* gene expression after oxidative stress (10 mmol L^−1^ H_2_O_2_). Relative expression was determined with reference to untreated control (corresponding to 1.0 at *Y* axis). The expression of genes was normalized with the *sigA* transcript level. The mean value and standard deviations were calculated from two different experiments and plotted for each set of data. (C) Western blot of SigF protein using protein samples from exponential and stationary phase cultures under treated (10 mmol L^−1^ H_2_O_2_) and untreated conditions. Apparently similar levels of SigF proteins were detected in treated and untreated samples. Gel picture shows equal loading of proteins.

But, similar to earlier findings (Provvedi et al. 2008), the *sigF* deletion mutant was found to be more susceptible than the wild type strain upon exposure to hydrogen peroxide mediated oxidative stress (Fig. 1A). Complemented strain (SFKO1/*sigF*) restored the survival after oxidative stress. Since, *sigF* was not found to be induced upon oxidative stress in previous studies (Singh and Singh 2008), we examined the *sigF* expression at RNA and protein level after treatment with hydrogen peroxide. No difference in the *sigF* expression level was noticed upon oxidative stress using log phase and stationary phase cultures (Fig. 1B and C). This suggests that SigF indirectly regulates H_2_O_2_ sensitivity in *M. smegmatis*.

### Loss of carotenoid pigment renders increased H_2_O_2_ sensitivity to the *sigF* mutant

**Figure 2:**
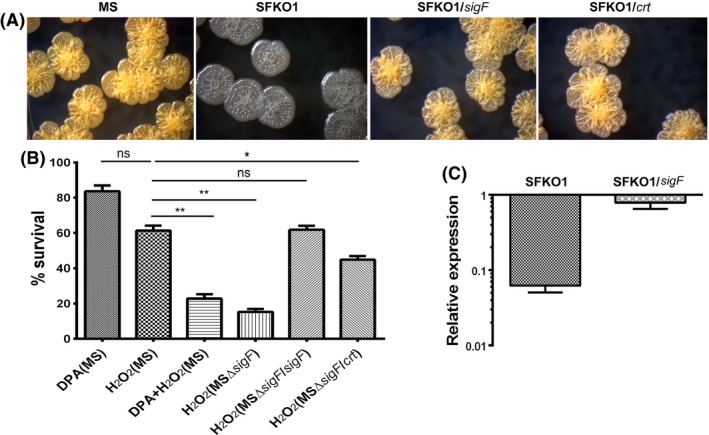
Complementation of Δ*sigF* mutant with *crt* locus genes restores the wild type phenotype. (A) Loss of pigmentation in Δ*sigF* mutant (SFKO1) is restored in *sigF* (SFKO1/*sigF*) and *crt* (SFKO1/*crt*) complemented strains, similar to *Mycobacterium smegmatis* WT (MS). (B) Treatment with DPA (0.1 mmol L^−1^) reduces survival of *M. smegmati*s WT (MS) cells to 80% with respect to untreated control (100%). DPA treated MS cells when exposed to H_2_O_2_ showed reduced survival which was relatively similar to H_2_O_2_ treated Δ*sigF* mutant cells and much lower than wild type treated cells. Susceptibility of Δ*sigF* mutant to oxidative stress is completely restored after complementation with *sigF* and nearly to a similar extent after complementation with *crt* locus genes. Data were collected from three different experiments. The mean values and standard deviations were plotted for each set of data. *P < 0.05, **P < 0.01 relative to H_2_O_2_ treated *M. smegmati*s WT (H_2_O_2_/MS) as determined by paired *t*‐test. (C) Expression of *crtI* gene in SFKO1. In complemented strain SFKO1/*sigF* expression was restored to almost wild type level. The expression of genes was normalized with the *sigA* transcript level. The mean value and standard deviations were calculated from two different experiments and plotted for each set of data.

Disparate response to oxidative stress was reported in saprophytic and pathogenic mycobacteria (Sherman et al. 1995). Saprophytes like *M. aurum* and *M. smegmatis* produce carotenoids, which are known scavengers of free radicals (Levy‐Frebault and David 1979) and enhance the strength of the cell wall due to their lipophilic nature and intercalation into the cell membrane (Kubler and Baumeister 1978). *M. smegmatis* mc^2^155 colonies produce pale yellow pigment (carotenoid isorenieratene) when incubated under light for 5–6 days. Deletion of *sigF* resulted in loss of pigmentation in SFKO1 (Fig. 2A) which was mostly restored after complementation with the *sigF* gene (SFKO1/*sigF*) (Fig. 2A), suggesting that the loss of pigmentation is specifically due to deletion of *sigF*. Because carotenoids are robust antioxidants and fortifiers of cellular barriers they are deemed beneficial for withstanding the stress beard by saprophyte like *M. smegmatis*. Since, we did not find the appreciable differences in the *sigF* expression after peroxide mediated oxidative stress despite the marked sensitivity of the Δ*sigF* mutant to H_2_O_2_, we reasoned that this phenotypic characteristic of the *M. smegmatis* Δ*sigF* mutant might be due to absence of carotenoids in the mutant. Moreover, the key detoxifying enzymes of reactive oxygen species in mycobacteria, *katG* and *ahpC* were found to be SigF independent (Gebhard et al. 2008; Hümpel et al. 2010). To test our hypothesis, we treated *M. smegmatis* mc^2^155 cells with diphenylamine (DPA), a known inhibitor of carotenogenesis in mycobacteria (Houssaini‐Iraqui et al. 1993), and subjected the DPA‐treated bacterial cells to hydrogen peroxide mediated oxidative stress. The DPA‐treated bacteria showed pronounced sensitivity to oxidative stress, comparable to *M. smegmatis* Δ*sigF* mutant strain (Fig. 2B). This was duly confirmed when SFKO1/*crt* strain apart from restoring the pigmentation (Fig. 2A) showed a significant recovery in survival following hydrogen peroxide mediated oxidative stress akin to SFKO1/*sigF* strain (Fig. 2B).

Carotene isorenieratene is the characteristic pigment of almost all orange‐pigmented mycobacteria including *M. phlei* (Goodwin and Jamikorn 1956, 1956), *M. aurum* (Levy‐Frebault and David 1979), *M. avium,* and *M. intracellulare* (Tarnok and Tarnok 1970, 1970). The synthesis of isorenieratene requires farnesyl pyrophosphate as a precursor, which leads to isorenieratene in five metabolic steps involving, CrtE, CrtB, CrtI, CrtY, and CrtU (Provvedi et al. 2008). It was postulated that in the absence of SigF, transcription of *crt* operon is off, hence SFKO1 mutant lacks pigmentation. Evidently, *crtI* transcript was found to be several‐fold downregulated in SFKO1 mutant in comparison to wild type strain (Fig. 2C) and the expression (Fig. 2C) as well as pigmentation (Fig. 2A) were restored, almost to the wild type level, in the complemented SFKO1/*sigF* strain. In *M. smegmatis* genome, a carotenogenic gene cluster comprises six open reading frames, *crtIBYcYdUV*, each transcribed in the same direction. The GGPP synthase gene, *crtE*, was found far away from the *crt* locus. The upstream regions of *crtI* gene harbored a canonical SigF promoter signature (Provvedi et al. 2008). When *crt* locus genes were overexpressed in SFKO1/*crt* strain, SFKO1/*crt* akin to SFKO1/*sigF*, restored the pigmentation (Fig. 2A) which was lost due to *sigF* deletion, suggesting that the SigF directly regulates the carotenoid biosynthesis and thereby the pigmentation of bacterial colonies in *M. smegmatis*. These results established that in *M. smegmatis* SigF confers resistance to hydrogen peroxide mediated oxidative stress largely through the carotenoid pigments.

### SigF modulates cell wall architecture by affecting GPL distribution and lipid biosynthesis

**Figure 3:**
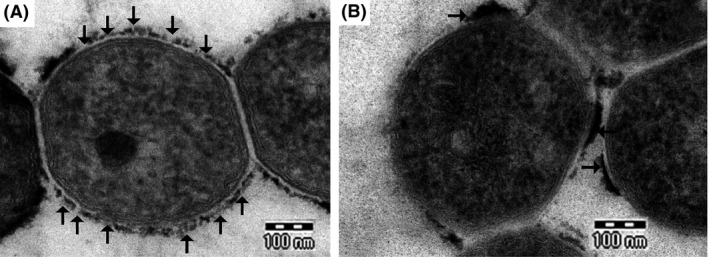
Transmission electron micrographs showing structure of cell envelope of *M. smegmatis* wild type (A) and Δ*sigF* mutant (B) strains. Note the even distribution of GPLs around wild type cells while distribution of GPLs is patchy in mutant cells.

**Figure 4:**
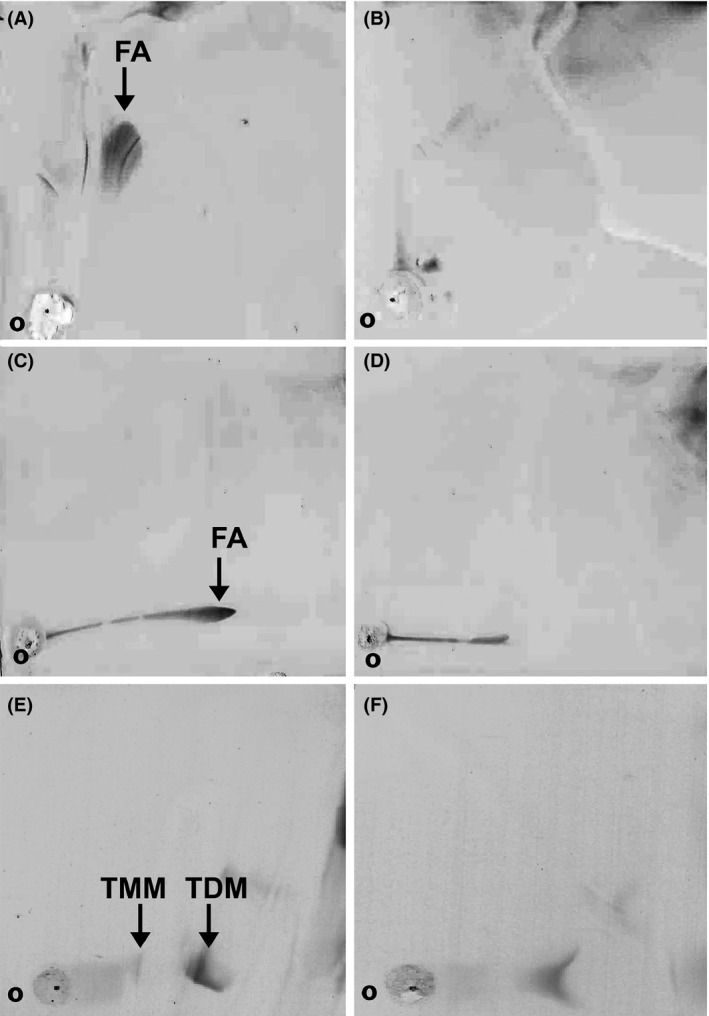
2D TLC analysis of nonpolar lipids from *Mycobacterium smegmatis* wild type (A, C, E) and Δ*sigF* mutant (B, D, F). Different solvent systems, described in methods, were used to develop TLC plates: A and B developed with solvent system B, C, and D developed with solvent system C, E, and F developed with solvent system D. The arrows indicate the missing fatty acids (FA) in Δ*sigF* mutant (B and D) and TMM (Trehalose monomycolate), TDM (Trehalose dimycolate) in panel F.

Previously, in *M. smegmatis*, we observed increased *sigF* expression upon exposure to isoniazid, ethambutol, and SDS (Singh and Singh 2008). Isoniazid and ethambutol specifically target cell wall biosynthesis process in mycobacteria, whereas SDS is an ionic detergent that affects the cell wall architecture. Overexpression of *sigF* in *M. tuberculosis* was reported to alter the regulation of many cell wall‐associated proteins, suggesting a role for SigF in maintaining cell wall architecture in mycobacteria (Forrellad et al. 2013). To examine the effect of *sigF* deletion on the cell wall architecture in *M. smegmatis*, we performed transmission electron microscopy using *M. smegmatis* WT and Δ*sigF* mutant cells. In *M*. *smegmatis*, GPLs constitute the major cell‐surface glycolipids and react with ruthenium red to give the electron‐dense appearance to the outermost cell envelope layer (Etienne et al. 2002). We noticed uniform distribution of GPLs on the surface of WT cells (Fig. 3A), while Δ*sigF* mutant cells displayed patchy GPLs distribution (Fig 3B). Next, we analyzed the total GPLs in wild type and Δ*sigF* mutant by TLC and mass analysis (see supplemental material, Fig. S2), but no difference was found in GPLs profile of Δ*sigF* mutant, suggesting that the uneven distribution of GPLs in the Δ*sigF* mutant cells is not due to difference in overall content and type of GPLs. Then, we examined the profiles of other cell wall lipids. TLC analysis of polar lipids also did not reveal any differences (data not shown), but nonpolar lipids showed distinct TLC profiles. Lipids spots present in wild type cells (Fig. 4A and C) were conspicuously missing in Δ*sigF* mutant cells (Fig. 4B and D). We also noticed distinct differences in trehalose containing lipids (Fig. 4E and F), an important component for cell wall integrity, indicating that the SigF alters the cell wall lipid composition by modulating the lipid biosynthesis pathway.

### Genome‐wide gene expression studies of *Mycobacterium smegmatis ΔsigF* mutant and wild‐type strains

A genome‐wide gene expression analysis of the *M. smegmatis* mc^2^155 WT and Δ*sigF* mutant strains was performed using Agilent microarray platform. SigF‐regulated genes during exponential phase and stationary phase were indentified, as described in the methods. Difference in the expression of a gene was calculated as the Δ*sigF* mutant to WT expression ratio and is expressed as fold‐change; only ≥ 2‐fold difference in the gene expression (*P* ≤ 0.05) was considered for analysis. Under these conditions, 142 genes in exponential phase and 158 genes in stationary phase were found to be significantly down‐regulated in the Δ*sigF* mutant. A large number of genes showed reduced expression in both exponential and stationary phase cells, and almost similar numbers of genes were found to be down‐regulated exclusively in exponential and stationary phase cells (Table 1). We also identified enhanced expression of 39 genes in exponential phase cells and 49 genes in stationary phase cells in Δ*sigF* mutant strain. The entire expression data can be found in Data set S1 in the supplemental material. To validate the microarray results, real‐time PCR was performed on few randomly selected genes from microarray data. Similar to microarray results, the selected genes showed reduced expressions in real‐time PCR experiment (see supplemental material, Fig. S3) as well.

**Table 1:** Genes with reduced expressions in Δ*sigF* strain

Locus	Description	Fold‐change Exponential/Stationary	SigF consensus	Position from start codon
**Commonly down‐regulated genes (*P* ≤ 0.05) in exponential and stationary phase**				
MSMEG_0266^a^	Arginine decarboxylase	−4.44/−5.90	GTCG‐N_17_‐GGGAT	160
MSMEG_0267^a^	Esterase	−5.49/−4.58	GTTT‐N_15_‐GGGTA	27
**MSMEG_0278**	Hypothetical protein	−2.22/−2.90	GGTT‐N_14_‐GGGCC	
**MSMEG_0280**	Alpha/beta hydrolase	−1.93/−4.30	GGTT‐N_14_‐GGGCC	158
MSMEG_0375	Phospholipase D family protein	−3.98/−2.91	GTTC‐N_19_‐GGGCA	192
MSMEG_0451^a^	Oxidoreductase, FAD‐linked	−4.69/−3.40	GTTC‐N_19_‐GGGCC	49
MSMEG_0521	Conserved hypothetical protein	−2.42/−1.76	GTTT‐N_16_‐GGGTA	10
MSMEG_0637	Iron‐sulfur binding oxidoreductase	−6.02/−3.33	GTCG‐N_14_‐GGGCA	548
MSMEG_0669	Hypothetical protein	−5.44/−2.52	GTTC‐N_14_‐GGGCC	661
MSMEG_0670^a^	FAD dependent oxidoreductase	−2.06/−3.17	GGTT‐N_16_‐GGGTA	9
MSMEG_0671^a^	S‐(hydroxymethyl) glutathione dehydrogenase	−3.75/−4.97	GTTT‐N_15_‐GGGTA	47
MSMEG_0672^a^	Conserved hypothetical protein	−1.73/−3.73	GTTT‐N_15_‐GGGTA	50
**MSMEG_0684**	Aldehyde oxidase and xanthine dehydrogenase	−5.15/−5.17	GTTG‐N_15_‐GGGTA	
**MSMEG_0685**	Oxidoreductase, molybdopterin‐binding subunit	−5.09/−5.49	GTTG‐N_15_‐GGGTA	
**MSMEG_0686** a	Oxidoreductase	−3.87/−3.26	GTTG‐N_15_‐GGGTA	8
**MSMEG_0696**	Alanine‐rich protein	−4.86/−5.90	GTTT‐N_16_‐GGGAA	
**MSMEG_0697** a	Integral membrane protein	−4.37/−4.66	GTTT‐N_16_‐GGGAA	58
MSMEG_1076^a^	Conserved hypothetical protein	−5.82/−2.58	GTTT‐N_16_‐GGGTA	50
MSMEG_1097^a^	Glycosyl transferase, group 2 family protein	−5.63/−5.01	GTGT‐N_15_‐GGGTT	11
MSMEG_1112^a^	Aconitate hydratase, putative	−5.96/−5.32	CGTT‐N_16_‐GGGAA	8
MSMEG_1131^a^	Tryptophan‐rich sensory protein	−5.33/−4.80	GTGT‐N_16_‐GGGTA	9
MSMEG_1315^a^	Transporter	−4.17/−2.56	GTTG‐N_17_‐GGGTA	11
MSMEG_1361	Alpha‐mannosidase	−2.20/−2.13	GTCG‐N_19_‐GGGTG	541
MSMEG_1605	PhoU	−2.50/−3.21	GTCC‐N_15_‐GGGTT	22
MSMEG_1758^a^	Hypothetical protein	−4.54/−2.95	GTTT‐N_16_‐GGGTA	8
**MSMEG_1766** a	Conserved hypothetical protein	−6.28/−6.24	GTTT‐N_16_‐GGGAA	32
**MSMEG_1767**	Conserved hypothetical protein	−5.70/−6.47	GTTT‐N_16_‐GGGAA	
**MSMEG_1768**	Conserved hypothetical protein	−5.50/−6.05	GTTT‐N_16_‐GGGAA	
**MSMEG_1769**	UsfY protein	−5.91/−4.14	GTTT‐N_16_‐GGGAA	
MSMEG_1770^a^	Conserved hypothetical protein	−5.89/−3.34	GTTT‐N_16_‐GGGCA	64
MSMEG_1771^a^	Methylase, putative	−6.17/−5.69	GTTT‐N_15_‐GGGTA	29
MSMEG_1772	Conserved hypothetical protein	−5.91/−6.43	GTTT‐N_15_‐GGGTA	696
MSMEG_1773^a^	Conserved hypothetical protein	−5.98/−4.09	GTTT‐N_15_‐GGGAA	11
MSMEG_1774^a^	Conserved hypothetical protein	−6.17/−4.13	GTTT‐N_16_‐GGGTA	64
MSMEG_1775^a^	Cytochrome P450 monooxygenase	−3.84/−3.01	GTTT‐N_15_‐GGGTA	9
MSMEG_1777^a^	UsfY protein‐	−4.98/−6.43	GTTT‐N_16_‐GGGTA	69
**MSMEG_1778**	Conserved hypothetical protein	−3.16/−5.02	GTTT‐N_15_‐GGGTA	
**MSMEG_1779**	Hypothetical protein	−3.64/−4.24	GTTT‐N_15_‐GGGTA	
**MSMEG_1780**	Hypothetical protein	−3.06/−4.79	GTTT‐N_15_‐GGGTA	
**MSMEG_1781**	Hypothetical protein	−6.29/−5.71	GTTT‐N_15_‐GGGTA	
**MSMEG_1782** a	Oxidoreductase, dehydrogenase/reductase	−5.87/−6.15	GTTT‐N_15_‐GGGTA	221
**MSMEG_1783**	Hypothetical protein	−3.48/−3.45	GTGT‐N_16_‐GGGTA	
**MSMEG_1784** a	Type I topoisomerase	−4.30/−3.46	GTGT‐N_16_‐GGGTA	183
MSMEG_1787^a^	RsbW protein	−3.10/−5.90	GTTT‐N_17_‐GGGTA	56
**MSMEG_1788**	Conserved hypothetical protein	−3.80/−3.37	GGTT‐N_15_‐GGGCA	32
**MSMEG_1789**	Conserved hypothetical protein	−6.02/−6.41	GGTT‐N_15_‐GGGCA	
**MSMEG_1790**	Conserved hypothetical protein	−5.79/−6.31	GGTT‐N_15_‐GGGCA	
MSMEG_1792^a^	Conserved hypothetical protein ‐	−3.47/−4.44	GGGT‐N_14_‐GGGCA	268
MSMEG_1794^a^	Dehydrogenase	−5.60/−5.72	GTGT‐N_17_‐GGGTA	15
MSMEG_1801^a^	Hypothetical protein	−1.69/−4.23	GGTG‐N_18_‐GGGAA	173
MSMEG_1802^a^	ChaB protein	−4.71/−4.70	GTTT‐N_16_‐GGGCA	63
MSMEG_1804	RNA polymerase sigma‐F factor	−5.96/−5.79	GTTT‐N_16_‐GGGCA	1001
MSMEG_1853^a^	Na+/H+ antiporter NhaA	−2.14/−2.15	GTTT‐N_15_‐GGGTA	99
**MSMEG_1950**	Conserved hypothetical protein	−5.96/−4.67	GTCG‐N_16_‐GGGCA	354
**MSMEG_1951**	Conserved domain protein	−5.70/−5.50	GTCG‐N_16_‐GGGCA	
**MSMEG_2112** a	Secreted protein	−2.09/−1.58	GTTT‐N_15_‐GGGTA	24
**MSMEG_2115**	Conserved hypothetical protein	−4.09/−5.02	GTTT‐N_15_‐GGGTA	
**MSMEG_2343**	Methylesterase (part of *crt* locus, 2343–2347)	−5.70/−5.37	GTTT‐N_16_‐GGGTA	
**MSMEG_2344**	Dehydrogenase	−5.01/−5.09	GTTT‐N_16_‐GGGTA	
**MSMEG_2345**	Lycopene cyclase	−5.62/−6.23	GTTT‐N_16_‐GGGTA	
**MSMEG_2346**	Phytoene synthase	−5.80/−6.21	GTTT‐N_16_‐GGGTA	
**MSMEG_2347** a	Phytoene dehydrogenase	−5.66/−4.48	GTTT‐N_16_‐GGGTA	97
MSMEG_2376	Conserved hypothetical protein	−4.29/−5.23	GTTC‐N_19_‐GGGCC	49
MSMEG_2415^a^	Hemerythrin HHE cation binding region	−1.45/−4.80	GTTG‐N_15_‐GGGTA	61
MSMEG_2594	Asparagine synthase (glutamine‐hydrolyzing)	−2.17/−3.76	CTTC‐N_15_‐GGGTG	321
MSMEG_2837^a^	Nitrate reductase NarB	−4.43/−3.16	GTTT‐N_16_‐GGGTA	42
MSMEG_2838	Conserved hypothetical protein	−3.91/−3.09	GTTT‐N_16_‐GGGTA	
MSMEG_2913^a^	Hydrolase	−5.87/−4.88	GTTT‐N_15_‐GGGTA	3
**MSMEG_2924**	Permease binding‐protein component	−5.78/−3.65	GTTT‐N_16_‐GGGTA	
**MSMEG_2925**	Permease membrane component	−5.77/−5.79	GTTT‐N_16_‐GGGTA	
**MSMEG_2926**	Glycine betaine/carnitine/choline transport	−5.77/−4.41	GTTT‐N_16_‐GGGTA	
**MSMEG_2927** a	ABC transporter, permease protein OpuCB	−4.94/−4.06	GTTT‐N_16_‐GGGTA	39
MSMEG_2958^a^	Conserved hypothetical protein	−3.79/−5.19	GTTC‐N_15_‐GGGTA	24
MSMEG_3022^a^	Transglycosylase associated protein	−5.76/−4.06	GTTT‐N_16_‐GGGTA	30
MSMEG_3083	Nucleoside‐diphosphate sugar epimerase	−1.75/−4.88	GCTT‐N_16_‐GGGTA	451
MSMEG_3141^a^	Conserved domain protein	−3.03/−1.90	GTGT‐N_16_‐GGGTA	29
MSMEG_3255^a^	DoxX subfamily, putative	−3.16/−5.18	GTTT‐N_15_‐GGGAA	36
MSMEG_3289^a^	gp61 protein	−5.33/−5.60	GTTT‐N_15_‐GGGTA	29
MSMEG_3304^a^	Succinate semialdehyde dehydrogenase	−4.71/−5.73	GTGT‐N_15_‐GGGTA	25
MSMEG_3310	Integral membrane protein	−3.40/−2.13	GTGT‐N_18_‐GGGCA	248
MSMEG_3311	Acyl carrier protein	−2.54/−2.83	GTCG‐N_17_‐GGGAA	255
MSMEG_3418	Conserved hypothetical protein	−3.73/−2.54	GTCG‐N_14_‐GGGTA	1115
MSMEG_3419	Hypothetical protein	−5.54/−4.43	GTCG‐N_14_‐GGGTA	58
MSMEG_3439^a^	Hypothetical protein	−5.55/−4.00	GTTT‐N_15_‐CGGTA	59
MSMEG_3443^a^	Hypothetical protein	−1.31/−3.85	GTTT‐N_15_‐GGGAT	45
MSMEG_3536^a^	Sugar transport protein	−3.75/−2.48	GTGG‐N_16_‐GGGTA	134
MSMEG_3673^a^	4‐alpha‐glucanotransferase	−1.35/−4.92	GTTT‐N_16_‐GGGCA	195
MSMEG_4707^a^	Nonhaem bromoperoxidase	−2.68/−1.51	GTTT‐N_15_‐GGGTA	35
MSMEG_4918^a^	1,4‐alpha‐glucan branching enzyme	−2.21/−2.54	GGTT‐N_15_‐GGGTA	172
**MSMEG_5188**	Caax amino protease family	−3.92/−3.01	GGTT‐N_16_‐GGGTA	
**MSMEG_5189** a	Oxidoreductase	−3.42/−3.69	GGTT‐N_16_‐GGGTA	25
MSMEG_5342	Conserved hypothetical protein	−5.55/−5.21	GTTT‐N_16_‐GGCTA	386
MSMEG_5399	ATP‐dependent DNA helicase RecQ	−3.01/−3.11	GTTT‐N_15_‐GGGTA	
MSMEG_5400	Dehydrogenase	−4.36/−2.19	GTTT‐N_15_‐GGGTA	
MSMEG_5401	Conserved hypothetical protein	−3.58/−5.87	GTTT‐N_15_‐GGGTA	
MSMEG_5402^a^	Dehydrogenase DhgA	−5.99/−4.80	GTTT‐N_15_‐GGGTA	8
MSMEG_5496	MscS Mechanosensitive ion channel	−3.78/−3.41	GTCT‐N_16_‐GGGGA	80
MSMEG_5540	Conserved hypothetical protein	−2.59/−2.34	GTTT‐N_17_‐GGGTA	792
MSMEG_5542	Transcriptional regulator, HTH_3 family	−4.82/−4.69	GTTT‐N_17_‐GGGTA	518
MSMEG_5543^a^	Hypothetical protein	−5.13/−5.91	GTTT‐N_17_‐GGGTA	77
MSMEG_5590	Carboxylate‐amine ligase	−5.48/−3.09	GTTT‐N_15_‐GGGCA	14
MSMEG_5605	Cytochrome bd ubiquinol oxidase, subunit I	−2.07/−3.47	GGTG‐N_19_‐GGGAA	73
MSMEG_5616	Glyoxalase/bleomycin resistance protein	−4.87/−1.79	GTTT‐N_15_‐GGGTA	647
MSMEG_5617^a^	Immunogenic protein MPT63	−3.63/−5.99	GTTT‐N_15_‐GGGTA	70
MSMEG_5799	Nucleoside‐diphosphate‐sugar epimerase	−4.69/−3.76	GTTC‐N_16_‐GGGAT	849
MSMEG_5826	Pyruvate decarboxylase	−3.78/−3.79	GTTG‐N_14_‐GGGCA	711
MSMEG_6211^a^	Hypothetical protein	−4.39/−4.12	GGTT‐N_15_‐GGGTA	9
MSMEG_6212^a^	Hemerythrin HHE cation binding domain	−5.43/−3.87	GTTT‐N_15_‐GGGTA	51
MSMEG_6213^a^	Manganese containing catalase	−4.18/−5.96	GTTT‐N_15_‐GGGTA	40
MSMEG_6232^a^	Catalase KatA	−5.95/−5.17	GTTT‐N_16_‐GGGAA	67
MSMEG_6305^a^	Conserved hypothetical protein	−5.04/−2.49	GTTT‐N_16_‐GGGCA	8
MSMEG_6354	Serine esterase, cutinase family	−4.67/−5.88	GGTG‐N_16_‐GGGAA	1058
MSMEG_6355	Hypothetical protein	−5.39/−4.85	GTTC‐N_16_‐GGGAC	19
MSMEG_6467^a^	Starvation‐induced DNA protecting protein	−5.72/−5.55	GTTC‐N_16_‐GGGCA	100
MSMEG_6501	Hypothetical protein	−3.17/−2.95	GTCG‐N_17_‐GGGCC	1008
**MSMEG_6514**	Trehalose synthase‐fused maltokinase	−1.98/−2.75	GTGT‐N_16_‐GGGTA	
**MSMEG_6515**	Trehalose synthase	−2.03/−2.58	GTGT‐N_16_‐GGGTA	10
**MSMEG_6606**	Hypothetical protein	−3.15/−2.45	GTTC‐N_14_‐GGGCA	
**MSMEG_6607**	Hypothetical protein	−3.51/−2.52	GTTC‐N_14_‐GGGCA	
**MSMEG_6608**	Hypothetical protein	−4.87/−2.97	GTTC‐N_14_‐GGGCA	
**MSMEG_6609**	Hypothetical protein	−5.21/−4.43	GTTC‐N_14_‐GGGCA	
**MSMEG_6610**	Protein of unknown function DUF58	−5.50/−2.49	GTTC‐N_14_‐GGGCA	
**MSMEG_6612**	ATPase, MoxR family	−6.20/−4.25	GTTC‐N_14_‐GGGCA	147
**MSMEG_6615**	Hypothetical protein	−5.45/−6.20	GTTT‐N_15_‐GGGTA	
**MSMEG_6616**	S‐(hydroxymethyl)glutathione dehydrogenase	−4.93/−4.93	GTTT‐N_15_‐GGGTA	32
MSMEG_6664	Methylenetetrahydrofolate reductase family	−5.52/−3.67	GTTT‐N_15_‐GGGAA	462
**MSMEG_6665** **a**	Integral membrane protein	−1.42/−4.25	GTTT‐N_15_‐GGGAA	8
**MSMEG_6667**	Conserved hypothetical protein	−4.80/−3.43	GTTT‐N_15_‐GGGAA	
MSMEG_6727	Amino acid permease‐associated region	−6.51/−6.32	GCTT‐N_15_‐GGGTA	56
MSMEG_6728	Conserved hypothetical protein	−5.57/−4.75	GTGG‐N_15_‐GGGTG	165
MSMEG_6730	Putative oxidoreductase YdbC	−2.93/−2.09	GTTG‐N_18_‐GGGTA	462
**MSMEG_6765**	ABC‐2 type transporter superfamily	−2.52/−3.69	GGTG‐N_18_‐GGGTA	
**MSMEG_6766**	ABC transporter, ATP‐binding protein	−3.91/−3.99	GGTG‐N_18_‐GGGTA	
**MSMEG_6767**	Mycocerosic acid synthase	−3.39/−2.59	GGTG‐N_18_‐GGGTA	58
MSMEG_6768^a^	Halogenase	−4.57/−5.79	GCTT‐N_16_‐GGGTA	9
MSMEG_6769	Transporter	−4.11/−2.93	GGTG‐N_16_‐GGGAT	649
MSMEG_6812	Major facilitator superfamily	−1.86/−2.46	GGTT‐N_14_‐GGGGA	22
**Genes exclusively down‐regulated in exponential phase (*P* ≤ 0.05)**				
MSMEG_0482	Dihydroxy‐acid dehydratase	−2.67/1.40		
MSMEG_0586	STAS domain, putative	−2.76/0.43		
MSMEG_0651	Putative conserved exported protein	−2.21/0.74	GTTC‐N_19_‐GGGTG	1171
MSMEG_0757	Hypothetical protein	−2.22/0.79		
MSMEG_1114	Short chain dehydrogenase	−2.07/1.31	GTCG‐N_19_‐GGGGA	155
MSMEG_1656	Exodeoxyribonuclease III	−2.34/−0.06	GTCG‐N_17_‐GGGCC	20
MSMEG_1912	Muconolactone delta‐isomerase 1	−3.08/−0.96	GCTT‐N_18_‐GGGCA	348
MSMEG_2024	Hydroxymethylglutaryl‐CoA lyase	−2.90/−0.07	GTCG‐N_17_‐GGGCC	66
MSMEG_2425^a^	Ammonium transporter	−2.17/0.13	GTTC‐N_17_‐GGGTA	238
MSMEG_3137	Oxidoreductase	−2.33/1.77	GTGG‐N_14_‐GGGGA	992
MSMEG_3401	LamB/YcsF family protein	−2.68/−0.38		
MSMEG_3402	Cytosine permease, putative	−2.39/0.65		
MSMEG_3403	Formamidase	−3.48/0.58	GGTT‐N_14_‐GGGTT	1004
MSMEG_3417	Conserved hypothetical protein	−4.63/−1.19	GTGG‐N_15_‐GGGTG	402
MSMEG_3541	Cytochrome C biogenesis protein	−4.19/0.11	GTTT‐N_14_‐GGGGA	676
MSMEG_3562	4‐carboxymuconolactone decarboxylase	−2.41/0.96		
MSMEG_3583	Monooxygenase	−2.72/0.51	GGTG‐N_14_‐GGGCC	470
MSMEG_3660	Conserved hypothetical protein	−2.33/0.91		
MSMEG_3927	Peptidase M52, hydrogen uptake protein	−3.34/1.03		
MSMEG_3928	[NiFe] hydrogenase, alpha subunit, putative	−2.49/1.28	GTCG‐N_14_‐GGGTG	345
MSMEG_3929	[NiFe] hydrogenase, delta subunit, putative	−2.51/0.76	GTTG‐N_16_‐GGGCC	150
MSMEG_3945	Universal stress protein family	−2.60/0.40	GGTG‐N_16_‐GGGCC	571
MSMEG_3983	L‐carnitine dehydratase	−2.35/1.16		
MSMEG_4329	Propionyl‐CoA carboxylase beta chain	−2.36/−0.49	GGTG‐N_16_‐GGGCC	1037
MSMEG_4424	Endoribonuclease L‐PSP	−3.48/1.03		
MSMEG_4618	Isochorismatase family protein	−3.08/0.79		
MSMEG_5100	Pyruvate ferredoxin/flavodoxin oxidoreductase	−3.82/0.72	GGTG‐N_15_‐GGGGA	361
MSMEG_5180	Conserved hypothetical protein	−2.41/−0.84	GTTG‐N_14_‐GGGTG	233
MSMEG_5341	Dipeptidyl aminopeptidase	−2.22/0.91		
MSMEG_5343^a^	Conserved hypothetical protein	−3.09/−1.07	GTTT‐N_16_‐GGCTA	35
MSMEG_5374	Glutamate‐ammonia ligase	−2.22/−0.03		
MSMEG_5559	Metabolite/sugar transport protein	−2.83/0.35	GTTT‐N_16_‐GGGTA	39
MSMEG_5623	L‐carnitine dehydratase	−3.24/1.20	GTTC‐N_15_‐GGGCA	51
MSMEG_5731	Transcriptional regulator, GntR family	−2.31/0.25	GTCT‐N_18_‐GGGAT	785
MSMEG_6507	Glycogen debranching enzyme GlgX	−2.27/0.93	GGTG‐N_14_‐GGGAT	656
MSMEG_6508	MarR‐family transcriptional regulator	−2.82/3.11	GCTT‐N_17_‐GGGCC	142
MSMEG_6528	Conserved hypothetical protein	−3.82/0.91		
MSMEG_6611	Hypothetical protein	−2.83/2.43		
MSMEG_6820	Acid phosphatase SurE	−3.26/−0.98	GTTG‐N_13_‐GGGTA	87
**Genes exclusively down‐regulated in stationary phase (*P* ≤ 0.05)**				
MSMEG_0195	Steroid monooxygenase	0.30/−2.66	GTTG‐N_16_‐GGGAT	403
MSMEG_0964	Pyridoxamine 5‐phosphate oxidase family	−0.42/−5.10	GTTT‐N_16_‐GGGCA	259
MSMEG_1196	SNF2 domain protein	0.05/−2.47		
MSMEG_1297	Hydroxydechloroatrazine thylaminohydrolase	−0.08/−2.82		
MSMEG_1658	Ribonuclease, putative	−0.54/−3.26	GTCT‐N_17_‐GGGTA	50
MSMEG_1803	RsbW protein	−1.23/−3.56	GTTT‐N_16_‐GGGCA	548
MSMEG_1807^a^	Acetyl‐/propionyl‐coenzyme A carboxylase	0.07/−2.38	GGTT‐N_17_‐GGGTA	294
MSMEG_2373	Acetolactate synthase, small subunit	0.15/−2.83	GTTG‐N_17_‐GGGCA	386
MSMEG_3082^a^	Heme‐binding protein	−0.47/−3.59	GCTT‐N_16_‐GGGTA	67
MSMEG_3157	Conserved hypothetical protein	0.70/−2.22		
MSMEG_3184	Malto‐oligosyltrehalose trehalohydrolase	−1.30/−3.83	GTGT‐N_15_‐GGGCA	409
MSMEG_3254	RDD family, putative	−0.96/−3.85	GTTT‐N_15_‐GGGAA	923
MSMEG_3273	Glutamyl aminopeptidase, M42 family	−0.57/−3.38	GCTT‐N_15_‐GGGCC	164
MSMEG_3322	Hypothetical protein	−0.46/−2.14		
MSMEG_3358	YaeQ protein	−0.61/−2.01		
MSMEG_3593	Protein of unknown function	−0.70/−4.74	GTTT‐N_14_‐GGGCA	987
MSMEG_4082	Monoxygenase	0.38/−2.17	GTTG‐N_14_‐GGGCC	1024
MSMEG_4355	Peptide ABC transporter, permease protein	−1.20/−3.44	GGTT‐N_15_‐GGGCC	13
**MSMEG_4356**	Inner membrane ABC transporter permease	−0.82/−3.24	GTTC‐N_14_‐GGGCC	139
**MSMEG_4357**	ABC transporter, ATP‐binding protein	−0.80/−3.48	GTTC‐N_14_‐GGGCC	
**MSMEG_4358**	D‐beta‐hydroxybutyrate dehydrogenase	−0.44/−2.91	GTTC‐N_14_‐GGGCC	
MSMEG_4428	Conserved hypothetical protein	1.01/−3.26		
**MSMEG_4531**	Sulfate ABC transporter, permease CysW	0.98/−3.99	GTCG‐N_15_‐GGGTT	
**MSMEG_4532**	Sulfate ABC transporter, permease CysT	1.10/−2.41	GTCG‐N_15_‐GGGTT	
**MSMEG_4533**	Sulfate‐binding protein	1.36/−2.58	GTCG‐N_15_‐GGGTT	756
MSMEG_4864	3‐ketosteroid dehydrogenase	−0.03/−2.49	GTTC‐N_18_‐GGGGA	81
MSMEG_4991	Hypothetical protein	−1.66/−6.01	GGTG‐N_17_‐GGGCC	47
MSMEG_4993	Hypothetical protein	−1.52/−4.00	GTGT‐N_19_‐GGGCA	408
MSMEG_5003	O‐methyltransferase, family	−0.05/−3.82		
MSMEG_5301	Transcriptional regulator	−0.19/−2.10		
MSMEG_5491	Putative acyl‐CoA dehydrogenase	0.51/−2.00	GTGT‐N_17_‐GGGTT	783
MSMEG_5606	Cytochrome bd‐I oxidase subunit II	−1.24/−3.75	GTTG‐N_14_‐GGGTT	625
MSMEG_5880	Nicotine dehydrogenase	0.78/−2.05	GCTT‐N_17_‐GGGAA	733
MSMEG_5936	Conserved hypothetical protein	−1.18/−3.80		
MSMEG_6151	Alpha/beta hydrolase fold‐1	−0.12/−2.26		
MSMEG_6210	Conserved hypothetical protein	−1.04/−3.38		
MSMEG_6541^a^	Anti‐sigma factor antagonist	−0.66/−3.63	GTTT‐N_15_‐GGGTA	282
MSMEG_6819	Conserved domain protein	−1.70/−4.01		
MSMEG_6822^a^	Beta‐lactamase	−0.28/−2.61	GTTT‐N_16_‐GGGTA	46

Fold‐change in expression – Δ*sigF* strain/wild‐type gene expression ratio in log2 scale. SigF consensus (GTTT‐N_(14–19)_ – GGGTA) was found in the upstream regions of majority of the down‐regulated genes. Locus IDs in bold refer to genes that are clustered as operon in the genome. SigF consensus in such cases was found either in ORFs of preceding genes or in far upstream of the first gene of the cluster, e.g. SigF consensus was present 97 bp upstream of MSMEG_2347, MSMEG_2343–MSMEG_2347 constitute *crt* locus. ^a^Genes found down‐regulated in Hümpel et al. (2010) as well as in this study.

The SigF promoter consensus in *M. smegmatis* was first identified *in silico* (Provvedi et al. 2008), and was later improved upon by experimental data (Gebhard et al. 2008; Provvedi et al. 2008; Hümpel et al. 2010). Using an improved SigF promoter consensus from later studies, 1200 bp upstream of the annotated start codon of the down‐regulated genes (Table 1) were visually checked for sequence similarities. We searched 1200 bp upstream sequence because several genes were arranged in gene clusters wherein the SigF consensus was found far upstream of the down‐regulated genes or even in the ORFs of the preceding genes. It may be noted that the canonical SigF promoter consensus was located more than 1000 bp upstream of the *sigF* gene in *M. smegmatis* genome (Gebhard et al. 2008). We reasoned that the SigF‐dependent genes are likely to be down‐regulated in both stages of growth. Notably, genes that showed reduced expressions commonly in exponential as well as stationary phase cells, most of them showed the presence of the SigF promoter consensus in their upstream regions (Table 1), suggesting that they are SigF‐dependent. Majority of genes that showed reduced expressions in this study were also reported to be down‐regulated by Humpel et al. (Hümpel et al. 2010). They identified the SigF promoter consensus in the upstream regions of transcriptional regulators, *sigH3* (MSMEG_0573), *whiB1* (MSMEG_1919), *whiB4* (MSMEG_6199), and *phoP* (MSMEG_5872), but the expressions of these genes were found unaltered in the Δ*sigF* mutant. In this study, using our selection criteria (≥2‐fold, *P* ≤ 0.05), we identified three transcriptional regulators; MSMEG_5542 (HTH3 family), MSMEG_5731 (GntR family), and MSMEG_6508 (MarR family) which showed reduced expression in exponential phase, and MSMEG_5542, MSMEG_5301 (TetR family) with reduced expression in stationary phase. Of these MSMEG_5542, 5731, 6508 were found to have SigF consensus in their upstream regions. It is likely that the down‐regulated genes which did not show SigF foot‐prints in their upstream regions are indirectly regulated by SigF‐dependent transcriptional regulators. Several of the exclusively down‐regulated genes from exponential and stationary phase cells also showed SigF promoter consensus in their upstream regions, while few of them were found lacking the consensus. Based on the SigF promoter sequences, identified from this study, we deduced a profile of the SigF promoter consensus (Table 1), which showed the similar occurrence of the nucleotides at a given position in the earlier reported SigF promoter signature (Hümpel et al. 2010).

### *Mycobacterium smegmatis* Δ*sigF* mutant phenotype and SigF regulon

The *M. smegmatis* Δ*sigF* mutant displayed notable phenotypes likes, loss of pigmentation, pronounced sensitivity to oxidative stress and alteration in the cell wall architecture due to patchy distribution of GPLs in the cell wall. Correlating the loss of pigmentation phenotype the expressions of carotenoid biosynthesis genes (MSMEG_2243–MSMEG_2247) were found to be down‐regulated during both growth stages (Table 1). The SigF promoter consensus was identified in the upstream of the cluster and the reduced expression of *crtI*, the first gene of the cluster, was validated by real time PCR (Fig. 2C). Complementation of the Δ*sigF* mutant restored the original phenotype (Fig. 2A).

Regarding the sensitivity to oxidative stress the expressions of key enzymes that detoxify reactive oxygen intermediates, *katG* and *ahpC*, were found unaltered in the mutant strain, suggesting these genes are not regulated by SigF. We demonstrated that the overexpression of *crt* locus genes largely restores the susceptibility of Δ*sigF* strain to oxidative stress. Moreover, several genes which could possibly render resistance to Δ*sigF* strain against oxidative stress were found to be SigF‐dependent and showed reduced expressions in both growth stages of Δ*sigF* strain. Two potential hydrogen peroxide detoxifying enzymes, exclusively present in *M. smegmatis*, a manganese containing catalase (MSMEG_6213) and a heme containing catalase KatA (MSMEG_6232), showed reduced expressions in both stages in present study as well as in earlier report (Hümpel et al. 2010). A starvation‐induced DNA protecting protein (MSMEG_6467) linked with oxidative stress resistance in bacteria (Gupta et al. 2002) showed reduced expression in both growth stages. *M. smegmatis* is a saprophyte and dehydrogenase activity is considered to be a good measure of microbial oxidative activity in saprophytes. Many genes (MSMEG_1794, MSMEG_5400, MSMEG_5402, MSMEG_0684) encoding for dehydrogenages and predicted to perform oxidoreductase activity (SmegmaList) were found to be SigF‐dependent and down‐regulated in both growth stages. These are likely to render susceptibility to the mutant strain toward oxidative stress.

In *M. smegmatis*, GPL biosynthesis gene cluster maps to a single locus of ~65 kb in the genome, containing nearly 30 ORFs that included genes for the synthesis as well as transport of GPLs (Ripoll et al. 2007). In the genome‐wide gene expression study (see supplementary Data set S1) no genes from GPL biosynthesis gene cluster showed altered regulation in the Δ*sigF* mutant strain. We also did not find the SigF consensus signature in the upstream regions of genes clustered at this locus. This was in line with our earlier observation wherein we did not notice any difference in GPLs profile of Δ*sigF* mutant. However, a complete analysis of polar and nonpolar lipids from Δ*sigF* mutants showed distinct differences in 2D‐TLC profile of nonpolar lipids in mutant strain. Concomitant with these findings trehalose biosynthesis genes (MSMEG_6514, MSMEG_6515) and mycocerosic acid synthase genes (MSMEG_6765 to MSMEG_6767) were found to be significantly down‐regulated in Δ*sigF* strain (Table 1). MSMEG_6515 encodes for trehalose synthase which enables the conversion of glycogen into trehalose. The SigF promoter consensus was identified in the upstream of these genes, indicating that trehalose and mycocerosic acid synthase (MAS) genes are directly regulated by SigF and affect the cell wall architecture by inhibiting lipid biosynthesis pathway in *sigF* mutant.

### Post‐translational regulation of SigF in *Mycobacterium smegmatis*: overexpression of *rsbW* mimics the *M. smegmatis* Δ*sigF* mutant phenotype

**Figure 5:**
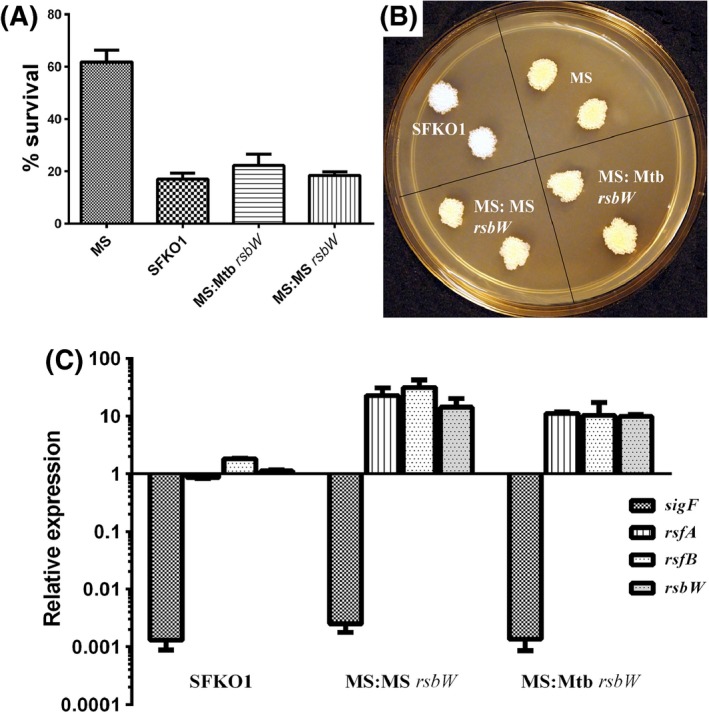
Increased susceptibility to oxidative stress (A) and loss of pigmentation (B) in *Mycobacterium smegmatis rsbW* overexpressing strain MS:MS *rsbW* and *M*. *tuberculosis rsbW* overexpressing strain MS:Mtb*rsbW*, nearly similar to Δ*sigF* mutant strain (SFKO1). (C) Relative expressions of *sigF*,* rsbW*,* rsfA,* and *rsfB* in SFKO1, MS:MS *rsbW* and MS:Mtb*rsbW* strains were determined from the RNA samples isolated from log phase cultures. The mRNA levels of *rsbW*,* rsfA,* and *rsfB* appear to be similar to wild type in SFKO1, while the *sigF* mRNA level is several‐fold reduced in SFKO1, MS:MS *rsbW* and MS:Mtb*rsbW* strains. The increased *rsbW* level in MS:MS *rsbW* and MS:Mtb*rsbW* strains amounts to the ectopically expressed *rsbW* under the *hsp60*_pr_ in these strains. *rsfA* and *rsfB* mRNA levels are also induced in both recombinant strains with respect to the wild type. Expression of genes was normalized with the *sigA* transcript level. The mean value and standard deviations were calculated from two different experiments.

Sigma factors activity is post‐translationaly regulated by their cognate anti‐sigma factors, which sequester them and make them unavailable for RNAP. In *M. tuberculosis*, SigF is post‐translationally regulated by its cognate anti‐sigma factor RsbW, which is, in turn, regulated by two anti‐anti‐sigma factors, RsfA and RsfB (Beaucher et al. 2002). Both are able to disrupt the RsbW‐SigF complex, releasing SigF to allow its association with RNA polymerase. In *M. smegmatis rsbW* (MSMEG_1803) is colocalized (Fig. S1) and cotranscribed with *sigF* (MSMEG_1804) (Gebhard et al. 2008). But, barring the sequence similarity with *M. tuberculosis* RsbW (Rv3287c), there has been no experimental evidence till date which demonstrates that MSRsbW binds to SigF and regulates it negatively. We argued that if MSMEG_1803 is indeed the anti‐*SigF*, RsbW, negatively regulating the SigF in *M. smegmatis*, overexpression of MSMEG_1803 in *M. smegmatis* wild type cells should sequester the prevailing pool of SigF and thereby making them unavailable for binding to RNA polymerase. This will impede the expression of SigF regulon and the MSMEG_1803 overexpressing *M. smegmatis* cells will produce a phenotype akin to *M. smegmatis* Δ*sigF* mutant. As shown in Fig. 5(A) and (B), we observed loss of pigmentation and increased susceptibility to oxidative stress in strain MS:MS*rsbW* nearly similar to SFKO1, the Δ*sigF* mutant strain. This proved that MSMEG_1803 indeed encodes for the cognate anti‐SigF protein which binds to SigF in *M. smegmatis* and regulates it negatively. Similar observations were made with *M. smegmatis* wild type cells overexpressing *M. tuberculosis rsbW* (MS:Mtb*rsbW*) (Fig. 5A and B), which further established that MSMEG_1803 is true ortholog of Mtb*rsbW*, as both strains produced similar phenotypes akin to SFKO1. To establish that the observed phenotypes of MS:MS*rsbW* and MS:Mtb*rsbW* strains are indeed due to overexpression of *rsbW* and sequestering of SigF proteins we performed real time semiquantitative RT‐PCR of these genes in *M. smegmatis* wild type, SFKO1 and overexpressing recombinant strains. We also examined the expression levels of two putative anti‐anti‐*sigF* proteins RsfA (MSMEG_1786) and RsfB (MSMEG_6127) from *M. smegmatis*, which were identified based on their homology to *M. tuberculosis* RsfA and RsfB. As observed in Fig. 5(C) the expression levels of *rsbW*,* rsfA,* and *rsfB* were found to be similar to wild type, while the *sigF* was nearly absent, owing to its deletion, in SFKO1 strain. However, the expressions of these genes were found to be similar in MS:MS*rsbW* and MS:Mtb*rsbW* strains, suggesting that MS*rsbW* (MSMEG_1803) is indeed similar to Mtb*rsbW*. A negligible expression of *sigF* gene was noticed in both strains, which implies that enhanced cellular level of RsbW protein, owing to its overexpression (Fig. 5C), completely sequestered the SigF protein, and, in turn shut down the expression of *sigF* gene, which is transcriptionally autoregulated. Since the *sigF* is cotranscribed with *rsbW* the increased *rsbW* level in MS:MS*rsbW* and MS:Mtb*rsbW* strains amounts to the ectopically expressed *rsbW* under the control of *hsp60*_pr_ in these strains. Interestingly, the expressions of *rsfA* and *rsfB* were also found to be induced, similar to *rsbW*, in both recombinant strains. RsfA and RsfB are known to antagonize RsbW, therefore, it is possible that some feedback machinery in the bacterial cell would have sensed the increased cellular level of RsbW and invoked an ensuing response by transcriptionally upregulating the expression of both anti‐*sigF* antagonists. It may be noted that the expression levels of RsfA (MSMEG_1786) and RsfB (MSMEG_6127) were not significantly altered in Δ*sigF* mutant strain in genome wide gene expression analysis performed in this study and by Hümpel et al. 2010. Also both these genes lacked SigF footprints in their upstream regulatory regions.

**Figure 6:**
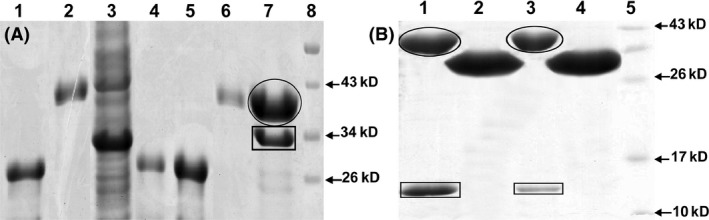
(A) *Mycobacterium smegmatis* SigF interaction with its anti‐sigma factor MSRsbW using pull‐down assay. Lanes: 1‐ purified GST, 2‐ purified GST‐MSRsbW, 3‐ overexpressed MSSigF, 4‐ GST protein with MSSigF (Eluted with 5 mmol L^−1^ RG), 5‐ GST protein with MSSigF (Eluted with 10 mmol L^−1^ RG), 6‐ GST‐MSRsbW with MSSigF (Eluted with 5 mmol L^−1^ RG), 7‐ GST‐MSRsbW (oval) with MSSigF (rectangle) (Eluted with 10 mmol L^−1^ RG), 8‐ Prestained protein marker. (B) *M. smegmatis* anti‐sigma factor antagonists, RsfA and RsfB, interactions with its anti‐sigma factor MSRsbW using pull‐down assay. Lanes: 1‐ GST‐MSRsbW (oval) with MSRsfA (rectangle) (Eluted with 10 mmol L^−1^ RG), 2‐ GST protein with MSRsfA (Eluted with 10 mmol L^−1^ RG), 3‐ GST‐MSRsbW (oval) with MSRsfB (rectangle) (Eluted with 10 mmol L^−1^ RG), 4‐ GST protein with MSRsfB (Eluted with 10 mmol L^−1^ RG), 5‐ prestained protein marker.

Furthermore, using bacterial two‐hybrid experiment we analyzed the interactions of *M. smegmatis* anti‐SigF RsbW with SigF and its two antagonists RsfA and RsfB. *M. smegmatis* RsbW showed very strong interactions with SigF and RsfA while a comparatively weak interaction was noticed with RsfB (Table 2). Similar results were obtained when we allowed *M. tuberculosis* RsbW to interact with *M. smegmatis* SigF, RsfA, and RsfB (Table 2). On the other hand, we did not notice any interaction when *M. smegmatis* RsbW was allowed to interact with *M. smegmatis* SigA, which confirmed the specificity of MSRsbW to its cognate sigma factor SigF. To further confirm these interactions we performed GST pull down assay. *M. smegmatis* RsbW was overexpressed as GST tagged protein (GST‐MSRsbW) using pET41a+ vector in *Escherichia coli*, purified and immobilized on GST beads. A column was prepared with GST‐MsRsbW immobilized beads and whole cell lysates of recombinant *E. coli* strains overexpressing *M. smegmatis* SigF, RsfA, and RsfB proteins were applied and allowed to bind to GST‐MsRsbW. Subsequently, interacting proteins were eluted using reduced glutathione and electrophoresed on SDS‐PAGE (Sodiumdodecyl sulfate polyacrylamide gel electrophoresis) (Fig. 6). Individual bands were excised and sequenced using MALDI/MS (data not shown). We noticed similar level of interactions between RsbW, SigF, RsfA, and RsfB proteins as it was observed in bacterial two‐hybrid assay. Thus, combined together, bacterial two‐hybrid and GST pull down results clearly established that MSMEG_1803 encodes for anti‐SigF RsbW protein in *M*. *smegmatis* which specifically and strongly interacts with its cognate sigma factor SigF and its antagonists RsfA and RsfB. The fact that these proteins showed similar level of interactions with *M. tuberculosis* RsbW suggests that most likely, similar to *M. tuberculosis*, in *M. smegmatis* SigF is post‐translationally regulated by its anti‐sigma factor RsbW, which is in turn regulated by its antagonists RsfA and RsfB. However, further experiments are required to elucidate the regulation of these interactions with respect to different physiological states of mycobacterial cells. It would be of interest to examine whether some more SigF antagonists are present in *M. smegmatis* genome as predicted by Hümpel et al. (2010) in their studies.

**Table 2:** Interactions of anti‐SigF (RsbW) with its antagonists (RsfA and RsfB) and SigF

Interacting proteins	
pBT‐LGF2 + pTRG‐GAL11^P^	+++
pBT + pTRG‐MS*rsbW* (MSMEG_1803)	−
pBT‐MS*sigA* + pTRG‐MS*rsbW*	−
pBT‐MS*sigF* + pTRG‐MS*rsbW*	++++
pBT‐MS*rsfA* + pTRG‐MS*rsbW*	++++
pBT‐MS*rsfB* + pTRG‐MS*rsbW*	++
pBT + pTRG‐Mtb*rsbW* (Rv3287c)	−
pBT‐MS*sigF* + pTRG‐Mtb*rsbW*	++++
pBT‐MS*rsfA* + pTRG‐Mtb*rsbW*	+++++
pBT‐MS*rsfB* + pTRG‐Mtb*rsbW*	++

Different levels of interactions are denoted as: <10% (−), 10–20% (+), 20–40% (++), 40–60% (+++), 60–80% (++++), >80% (+++++). Control vectors carrying bait protein pBT‐LGF2 and target protein pTRG‐GAL11P showed strong (+++) interaction and considered as reference.

## Conclusions

In this study, we report that in *M. smegmatis* the SigF is not essential for growth of bacterium. Deletion of *sigF* results in loss of carotenoid pigmentation which rendered increased susceptibility to H_2_O_2_ induced oxidative stress as complementation of Δ*sigF* mutant with carotenoid genes largely restores the phenotype. In *M. smegmatis*,* sigF* deletion altered the outer most layer of the cell envelope and the cell wall lipid composition by modulating the lipid biosynthesis pathway. *M. smegmatis* SigF regulon included variety of genes expressed during exponential and stationary phases of growth and those responsible for oxidative stress, lipid biosynthesis, energy, and central intermediary metabolism. We report the identification of a SigF antagonist, an anti‐sigma factor (RsbW), which upon overexpression in *M. smegmatis* wild type cell produced a phenotype similar to *M. smegmatis* Δ*sigF* mutant. Two anti‐sigma factor antagonists, RsfA and RsfB are also identified and their interactions with anti‐sigma factor were confirmed using bacterial two‐hybrid and GST pull down.

## Experimental Procedures

### Bacterial strains and culture conditions

Bacterial strains and plasmids used in this study are described in Table 3. *M. smegmatis* mc^2^155 wild type and derivative strains were grown at 37°C in Middlebrook 7H9 (Difco) liquid culture medium supplemented with 10% albumin‐dextrose‐catalase (ADC), 0.5% glycerol, and 0.05% Tween‐80 or on Middlebrook 7H10 (Difco) solid culture medium supplemented with 10% oleic acid‐albumin‐dextrose‐catalase (OADC) and 0.5% glycerol. *E. coli* cultures were grown in Luria‐Bertani (LB) broth with the addition of ampicilin (100 *μ*g mL^−1^), kanamycin (50 *μ*g mL^−1^), and hygromycin (100 *μ*g mL^−1^), as required.

**Table 3:** Bacterial strains and key plasmids used in this study

Strains or plasmids	Relevant properties	Reference or source
*Escherichia coli* strains		
*E. coli* DH5*α*	F^‐^ *φ*80*lac*ZΔM15 Δ(*lac*ZYA‐*arg*F)U169 *rec*A1 *end*A1	Invitrogen
XL1‐Blue MRF′	*rec*A1 *gyr*A96 *rel*A1 *lac* [F′*pro*AB *lac*Iq*Z*Δ*M*15 Kan^r^]	Agilent Technologies
XL1‐Blue	*rec*A1 *gyr*A96 *rel*A1 *lac* [F*′ lac*Iq *HIS*3 *aadA* (Kan^r^)]	Agilent Technologies
pLYSY^+^	*lac*Iq(Cam^r^)*/fhu*A2 *lacZ*::T7* gene*1 *end*A1	New England Biolabs
*E. coli* C41	Expression vector	Novagen
Mycobacterial strains		
*Mycobacterium smegmatis* ATCC607	*M. smegmatis* parent strain of mc^2^155	Late Jean‐Mark Reyrat, University of Paris
*M. smegmatis* ATCC607 Δ*sigF*	*sigF* deleted ATCC607 strain, Str^r^	
*M. smegmatis* mc^2^155	High transforming *M. smegmatis* strain	Departmental Stock
SFKO1	*sigF* deleted mc^2^155 strain, Hyg^r^	This study
SFKO1/*sigF*	mc^2^155 Δ*sigF* mutant complemented with *sigF*	This study
SFKO1/*crt*	mc^2^155 Δ*sigF* mutant complemented with *crt*	This study
MS:MS*rsb*W	mc^2^155:*hsp60*pr‐MS*rs*W, Km^r^	This study
MS:Mtb*rsb*W	mc^2^155:*hsp60*pr‐Mtb*rs*W, Km^r^	This study
*M. tuberculosis* H37Rv	Laboratory strain of tubercle bacilli	Departmental Stock
Plasmids		
pDrive	PCR cloning vector, Amp^r^, Km^r^	Qiagen, India
pTZ57R/T	PCR cloning vector, Amp^r^,	Fermentas, India
pMV261	*E. coli*‐mycobacterial shuttle vector, Km^r^	Stover et al. (1991);
pMV306	Mycobacterial integrative vector, Km^r^	Stover et al. (1991)
pET28a, 41a(+)	Expression vectors, Km^r^	Novagen
pTZ*sigF*1	pTZ carrying MS *sigF* ORF at NcoI‐HindIII	This study
pTZ*sigF*2	pTZ carrying MS *sigF* flanked by XbaI‐BamHI	This study
pET*sigF*	pET28a carrying MS *sigF* at NcoI‐HindIII	This study
pDΔ*sigF*	pDrive carrying *sigF* allelic exchange cassette, hyg^r^	This study
pMV306*sigF*	pMV306 containing *hsp60pr‐sigF* at NotI‐HindIII	This study
pMV306*crt*	pMV306 carrying *crt* locus at XbaI‐HindIII	This study
pTRG‐MS *rsbW*	pTRG vector carrying MS *rsbW* ORF at EcoRI‐XhoI, Tet^r^	This study
pTRG‐Mtb *rsbW*	pTRG vector carrying Mtb *rsbW* ORF at EcoRI‐XhoI, Tet^r^	This study
pBT‐MS *sigF*	pBT vector carrying MS *sigF* ORF at EcoRI‐XhoI, Chl^r^	This study
pBT‐MS *sigA*	pBT vector carrying MS *sigA* ORF at EcoRI‐XhoI, Chl^r^	This study
pBT‐MS *rsfB*	pBT vector carrying MS *rsfB* ORF at EcoRI‐XhoI, Chl^r^	This study
pBT‐MS *rsfA*	pBT vector carrying MS *rsfA* ORF at EcoRI‐XhoI, Chl^r^	This study
pBT‐LGF2	Two hybrid interaction control bait plasmid	Agilent Technologies
pTRG‐Gal11^P^	Two hybrid interaction control target plasmid	Agilent Technologies
pET41a‐MS *rsbW*	Expression vector carrying MS *rsbW* ORF at speI‐XhoI, Km^r^	This study
pET28a‐MS *rsfA*	Expression vector carrying MS *rsfA* ORF at NdeI‐XhoI, Km^r^	This study
pET28a‐MS *rsfB*	Expression vector carrying MS *rsfB* ORF at NdeI‐XhoI, Km^r^	This study
pMV261‐MS *rsbW*	pMV261 vector carrying MS *rsbW,* Km^r^	This study
pMV261‐Mtb *rsbW*	pMV261vector carrying Mtb *rsbW,* Km^r^	This study

Amp^r^, ampicillin resistant; Km^r^, kanamycin resistant; hyg^r^, hygromycin resistant; Tet^r^, tetracycline resistant; Chl^r^, chloramphenicol resistant; Str^r,^ streptomycin resistant.

### DNA manipulation, construction of sigF mutant, and its complementation

Recombinant DNA techniques were performed as per standard procedures (Sambrook et al., 2001) using *E. coli* DH5*α* as the initial host. Restriction and DNA modifying enzymes were obtained from Fermentas. Primers used in this study are described in Table 4. Preparation of electrocompetent cells and electroporation were done as previously described (Singh and Singh 2008). *M. smegmatis* mutant lacking *sigF* was constructed using allelic exchange method. For this, a hygromycin resistance cassette flanked by nearly 1 kb flanking regions of each side of the *sigF* gene was cloned into pDrive plasmid vector generating pDΔ*sigF*. The final allele exchange cassette contained 5′flank/Hyg^r^/3′flank in pDΔ*sigF*. 5′ and 3′ flanking regions contained a few nucleotide sequences of *sigF* gene which was later used for PCR amplification of *sigF* ORF from wild type and Δ*sigF* mutant. pDrive contains only *E. coli* origin of replication and, therefore, fails to multiply in mycobacteria and serves as suicide vector in mycobacteria. pDΔ*sigF* was electroporated into *M. smegmatis* mc^2^155 and transformants were selected on hygromycin (50 *μ*g mL^−1^) plates. The expected double cross‐over event would exchange *sigF* gene with hygromycin resistance marker in mutant strain. Selected colonies were first screened by PCR using MSSF1 and MSSF2 primers followed by sequencing and finally validated using Southern blotting. Southern blot was carried out using SmaI digested genomic DNA of *M. smegmatis* wild type and putative *sigF* deletion mutants using two probes, one specific for *sigF*‐*rsbW* (Probe 1) and another for *hyg* (Probe 2) (Fig. S1). The probe was labeled using Dig High Prime DNA labeling Kit (Roche) as per manufacturer's instructions. Firstly, probe‐1 corresponding to *sigF*‐*rsbW* was hybridized, signals were developed and then after deprobing the blot was rehybridized with probe‐2 corresponding to hygromycin. Blots were developed using chemiluminescence based detection kit (Roche) according to the manufacturer's instructions. The confirmed Δ*sigF* mutant strain is designated as SFKO1. For complementation of the Δ*sigF* mutant, *M. smegmatis sigF* ORF was PCR amplified and cloned into the NotI‐HindIII sites of the PMV306 (Stover et al. 1991), an integrative *E. coli*/mycobacterial shuttle vector, downstream to *hsp60* promoter to create pMV306*sigF*. Transformation of SFKO1 strain with pMV306*sigF* resulted in strain SFKO1/*sigF*. Similarly, *crt* locus genes were PCR amplified and cloned into the XbaI‐HindIII sites of the PMV306 at the downstream of *hsp60* promoter to create pMV306*crt* and SFKO1was transformed with pMV306*crt* to generate strain SFKO1/*crt*.

**Table 4:** Primers used in this study

Primers	Sequence (5′ to 3′)
MSSF1	TCTAGAGTGACGTCGGAATACGCAG
MSSF2	AAGCTTCTACTGCAGCTGGTCGCGCA
pETSF1	ACCATGGGCCATCATCACCACCAT
pETSF2	CACCACCATCATATGACGTCGGAA
SFAE1	AAGCTTATGCGGCGCATGG
SFRT3	AGGCACCGCTCGACGATCTTC
MSF3′F	TCTAGAGCGCACCGTGCTGGTGCTGC
MSF3′R	GATCCTGTCGTGGGATCGTGCGAGAG
PhytoFR1F	ACTAGTCTAGAATGAGCCGCGCGATCCCGCGAC
PhytoFR2R	ACTAGAAGCTTCGCCGCCACCGGCGGTGTGGTG
Real time	
MysART4	CATCTCGCTGGACCAGAC
MysART6	TGCAGCAGCGTGAACGACAC
SFRT1	GTGACGTCGGAATACGCAGACG
SFRT2	TCCGAGCCGCAAGTGGAGTTCC
MS 1802F	GGTCGGCAGAGGGAGTCGAC
MS 1802R	TTCTCGATAGCGGTCACCAG
MS 0670F	CCTACTCCACTTTCACATTC
MS 0670R	TACTGCATACCGGTGGCGAG
MS 1782F	TGGACTCCTTCGAATCCGAC
MS 1782R	GGTTTGTCGGCCATGTCCTC
MS 2594F	GCCATGGCAGAGACGATGTC
MS 2594R	CCGCTTCGGTCAGATCAATG
MS 6727F	TCATCCTCGGCGACGTGCTC
MS 6727F	GTGAGCAGGGCCAACATCAG
MS 1769F	TGACGAACCTGTCGATCATG
MS 1769R	ACCAGGCTGCTCACGAACAC
MS 6232F	ACCGTGACGTGCTGACCGAC
MS 6232R	TCTTCTCCAGGAAGTGGTAG
MS 2837F	CGCAACGTGTCGATCGATAC
MS 2837F	ACGATGCGTCCGTCCTTGAC
MS 2347F	GGCGGTTACCGGATCGACAC
MS 2347R	GGGAGCAACTGCAGGCGGTC
1803RTF	GAAACACCCGCTCGGGGCGA
1803RTR	CGTCGAAGTCGAGGTCCTCGA
Ms*rsfA*RTF	CAGCGTTGCCAAGAGGAGTA
MS*rsfA*RTR	TGGAGGCATCCAGGTCGCCG
MS*rsfB*RTF	CGAGCCAGGACCCGGCGAA
MS*rsfB*RTR	GGAACCGATCGCGTCTTCGA
Two Hybrid	
MS *sigA*1	ACGAATTCGTGGCAGCGACAAAGGCA
MS *sigA*2	GCACTCGAGCTAGTCCAGGTAGT
MS *rsbW*1	CTGGAATTCAGATGGCGGAAACACC
MS *rsbW*2	CTGCTCGAGTCACCGCAGCAGGC
Mtb *rsbW*1	CTGGAATTCAGATGGCCGACTCGG
Mtb *rsbW*2	GCACTCGAGTCACCTGCTGGATG
MS *sigF*1	AGTGAATTCCATGACGTCGGAATAC
MS *sigF*2	GCACTGGAGCTACTGCAGCTGGTC
MS *rsfB*1	TGAGAATTCCATGACGAGCCAGGAC
MS *rsfB*2	AGTCTCGAGTTATGTCTTCAACGACG
MS *rsfA*1	GGAATTCATGCCCACAATCAGCG
MS *rsfA*2	AGTCTCGAGCTAGGTGTTCTCCACC
Pull down	
MS *rsbW*3	CAACTAGTATGGCGGAAACACCCG
MS *rsbW*2	CTGCTCGAGTCACCGCAGCAGGC
Mtb *rsbW*3	CAACTAGTATG GCCGACTCGGATT
Mtb *rsbW*2	GCACTCGAGTCACCTGCTGGATG
MS *rsfA*HF	AGCCATATGCCCACAATCAGCGTTGC
MS *rsfA*HR	TCACTCGAGCTAGGTGTTCTCCACCAG
MS *rsfB*HF	AGCCATATGACGAGACCAGGACCCGGCGA
MS *rsfB*HR	TCACTCGAGTTATGTCTTCAACGA
pETSF1	ACCATGGGCCATCATCACCACCAT
pETSF2	CACCACCATCATATGACGTCGGAA

Restriction sites relevant to procedures used in this work are underlined.

### Susceptibility of *Mycobacterium smegmatis* strains to oxidative stress

For stress experiments, different *M. smegmatis* strains were grown to 0.6–0.8 OD_600_ (exponential phase) and 2.6–2.8 OD_600_ (stationary phase) and then cultures were split into aliquots. For oxidative stress, cultures were treated with H_2_O_2_ (10 mmol L^−1^), allowed to grow for 4 h at 37°C and plated thereafter in duplicates following 10‐fold serial dilution for CFU analysis. Untreated cultures were taken as control for stress experiments. The total number of colonies that appeared in the untreated control was considered 100%. Data were collected from three different experiments. The mean values and standard deviations were plotted for each set of data.

For inhibition of carotenoid biosynthesis, initially the dose of diphenylamine (DPA) was set so that ≥ 80% of *M. smegmatis* mc^2^155 wild type cells survive after DPA treatment. 0.1 mmol L^−1^ DPA treatment for 4–6 h ensured the survival of 80% wild type cells. Further experiments with different *M. smegmatis* strains (Fig. 2) were performed with exponentially grown culture at similar OD values (0.6–0.8). Cultures were incubated with 0.1 mmol L^−1^ DPA for 2 h before H_2_O_2_ treatment and stress susceptibility was analysed as described above.

### Generation of anti‐SigF antibody and immunodetection of SigF

The *M. smegmatis sigF* ORF was amplified using gene‐specific primers and cloned into PCR cloning vector pTZ57R/T. The clone was verified by DNA sequencing following which the ORF was relocated to the pET28a+ expression vector generating pETSigF. SigF was overexpressed as N‐terminal His_6_‐tagged recombinant in *E. coli* C41 cells, purified using Ni–NTA affinity chromatography and the purified His_6_‐SigF was used to raise anti‐SigF antibody in female New Zealand white rabbit, as described previously (Biswas et al. 2013). Immunodetection was performed with the primary antibody (polyclonal sera at 1:2000), followed by washing and incubation with the secondary antibody (anti‐rabbit IgG horseradish peroxidase conjugate at 1:40,000). The blots were developed using the chemiluminescent substrate (Pierce) and the signals were captured on the Bio‐Rad Chemidoc system.

### Transmission electron microscopy

Electron microscopy samples were prepared as described previously (Paul and Beveridge 1992). Briefly, fully grown cultures of *M. smegmatis* strains were diluted (1:100) in fresh LBGT broth and allowed to grow till 0.5 OD_600_. Cultures were centrifuged at 400 × g for 2 min to separate homogenous cell suspension from cell aggregates. Homogenous suspensions were transferred to new tubes and cells were harvested by centrifugation at 2600 × g for 5 min. Cells were washed five times with 0.1 mol L^−1^ cacodylate buffer (pH 6.8) and pellets (~50 mg wet weight) were fixed in 2.5% (w/v) glutaraldehyde, 0.05% ruthenium red in 0.1 mmol L^−1^ cacodylate buffer in dark at 4°C overnight. Cells were collected by centrifugation, washed thrice in 0.1 mol L^−1^ cacodylate buffer before fixing for 2 h in dark in 1% (w/v) osmium tetroxide, 0.05% ruthenium red in 0.1 mol L^−1^ cacodylate buffer. After this cells were washed thrice in 0.1 mol L^−1^ cacodylate buffer for 5 min each and embedded in 2% agarose gel. Blocks were dehydrated through a graded ethanol series of 20, 40, 60, 80, and 95% for 5 min each followed by two 10 min washes in absolute ethanol. Samples were embedded in EPON 812 resin at 60°C for 48 h. Ultra thin sections (50–70 nm) were obtained using Ultracut Ultra Microtome (Leica) and picked upon 200 mesh copper grids. Sections were poststained with uranyl acetate and Reynold's lead citrate. Microscopy was performed on a Philips FEI Technai‐12 Twin Transmission Electron Microscope and images were recorded using a SIS mega View II CCD camera attached with the microscope.

### Extraction and analysis of GPLs and total lipids from *Mycobacterium smegmatis*

GPLs extraction and analysis were performed as described earlier (Vats et al. 2012). The *M. smegmatis* wild type and mutant strains were grown in Middlebrook 7H9 medium supplemented with 10% ADC till late stationary phase (2.8–3.0 OD_600_). GPLs were extracted with CHCl_3_/CH_3_OH (2:1) at room temperature for 24 h. The supernatant was dried using rotatory evaporator till dryness. The lipid extract was deacetylated by 0.2 mmol L^−1^ NaOH in methanol at 37°C for 1 h followed by neutralization with glacial acetic acid. After drying, lipids were dissolved in CHCl_3_/CH_3_OH (2:1), spotted onto the TLC plate (Aluminium baked silica gel 60 F254) (Merck) and developed in CHCl_3_/CH_3_OH/H_2_O (90:10:1) solvent. GPLs were visualized by spraying with 5% *α*‐naphthol/sulfuric acid in ethanol followed by charring at 120°C for 10 min. The four de‐*O*‐acetylated GPLs (dGPLs) were named dGPL I, II, III, and IV, starting from the solvent front. For mass analysis GPLs were analysed and identified by ESI‐Q‐TOF‐MS (Absciex). [M+Na]^+^ ions of deacetylated GPLI, GPLII, GPLIII, and GPLIV were observed at *m/z* 1187, 1173, 1173, and 1159 respectively (Khoo et al. 1995; Vats et al. 2012).

Extractions and analysis of lipids were performed as described earlier (Slayden and Barry 2001). Lipids were extracted from freeze dried stationary phase grown *M. smegmatis* cells. Bacterial cells were resuspended in equal volume of methanolic saline and petroleum ether, mixture was stirred for 12–16 h and then allowed to separate following which nonaqueous phase containing the nonpolar lipids were removed and stored. An equal volume of petroleum ether was added to lower aqueous phase, mixture was stirred for 2 to 4 h, nonaqueous layer was removed and pooled with the first one. Nonpolar lipids were dried using a rotatory evaporator and resuspended in dichloromethane. Extraction of polar lipids was performed by adding chloroform (CHCl_3_), CH_3_OH, and 0.3% aqueous NaCl (9:10:3) to the extract. The entire mixture was stirred for 4 h and the solvent extract was separated from the biomass. Furthermore, the residues were extracted with CHCl_3_, CH_3_OH, and 0.3% aqueous NaCl (3:10:4) for 4 h. The polar lipid extracts were mixed with CHCl_3_ and 0.3% aqueous NaCl in equal ratio and the lower organic layer was separated discarding the upper aqueous layer. Polar lipids were dried using rotatory evaporator and resuspended in CHCl_3_ and CH_3_OH (2:1). 100 *μ*g of lipid extracts were spotted onto the TLC plate (aluminium baked silica gel 60 F254) (Merck) and developed using solvent systems described below. Lipids were detected by charring with 5% phosphomolybdic acid (MPA, Sigma‐Aldrich) in ethanol.

The solvent systems for 2D‐TLC: **System A:** (1) petroleum ether/ethyl acetate (98:2, three times) (2) petroleum ether/acetone (98:2). **System B:** (1) petroleum ether/acetone (92:8, three times) (2) toluene/acetone (95:5). **System C:** (1) chloroform/methanol (96:4) (2) toluene/acetone (80:20). **System D:** (1) chloroform/methanol/water (100:14:0.8) (2) chloroform/acetone/methanol/water (50:60:2.5:3). **System E:** (1) chloroform/methanol/water (60:30:6) (2) chloroform/acetic acid/methanol/water (40:25:3:6).

### Protein‐protein interaction analyses using bacterial two‐hybrid

BacterioMatch II two‐hybrid system (Agilent Technologies) was used for analyses of protein‐protein interactions. The system utilizes a double *HIS*3‐*aadA* reporter cassette which identifies interacting partners with plausibly reduced background. Detection of protein‐protein interactions is based on transcriptional activation of the *HIS3* reporter gene, which allows growth in the presence of 3‐amino‐1, 2, 4‐triazole (3‐AT), a competitive inhibitor of His3 enzyme. Positives are reconfirmed by using the *aadA* gene, which confers streptomycin resistance, as a secondary reporter.

*Mycobacterium smegmatis sigF*,* sigA*, anti‐*sigF rsbW* (MSMEG_1803), and anti‐*sigF* antagonists, *rsfA* (MSMEG_1786) and *rsfB* (MSMEG_6127) were amplified using gene specific primers (Table 4) and cloned into bait vector pBT at given enzyme sites (Table 3). Similarly, anti‐sigma factors from *M. smegmatis* (MS*rsbW*) and *M. tuberculosis* (Mtb*rsbW*) were amplified using gene specific primers (Table 4) and cloned into target vector pTRG at given enzyme sites (Table 3). All cloning steps were performed in *E. coli* XL1Blue strain, and the clones were verified by restriction digestion and DNA sequencing. To analyze interactions between two proteins, plasmid pairs carrying ORFs in pBT and pTRG vectors were cotransformed in XL1Blue derived reporter strain, provided with two‐hybrid system. Cotransformants were selected on M9 and M9‐3AT plates. The cotransformant containing pBT‐LGF2 and pTRG‐GaL11^P^ (Agilent) was used as a positive control for expected growth on the selective screening medium (M9 with 5 mmol L^−1^ 3‐AT). A cotransformant containing the empty vectors pBT and pTRG was used as a negative control. Further positives were verified using second reporter gene (*aadA)*, conferring streptomycin resistance. The interaction between the bait and target proteins was revalidated by patching cells from a putative positive colony from a selective screening medium (M9‐3AT) plate onto a dual selective screening medium (M9‐3AT + streptomycin 15 *μ*g mL^−1^) plate. CFU obtained on the nonselective screening medium (M9 without 3AT) and selective medium (M9‐3AT) plates were counted, and values were used to determine the percent interaction. The average and standard deviations were determined from data generated from two different experiments.

### Cloning, expression, purification of RsbW, SigF, RsfA and RsfB and GST pull down assay

*Mycobacterium smegmatis rsbW* ORF was amplified using gene specific primers and cloned into pET41a+ at SpeI and XhoI sites to generate pET41a‐MS*rsbW*. This allowed MS*rsbW* to be cloned in fusion with GST at its N‐terminal. Positive clones were verified by restriction digestion and DNA sequencing. Recombinant pET41a‐MS*rsbW* and pET41a+ plasmid carrying GST were separately transformed into *E. coli* pLysY^+^ cells and the transformants were selected on kanamycin. Selected colonies were allowed to grow till 0.6 OD_600_ and induced with 1 mmol L^−1^ IPTG at 30°C with continuous shaking for 4 h. Cells were pelleted by brief centrifugation and washed with cold PBS. The pellet was resuspended in buffer (50 mmol L^−1^ Tris pH 7.2, 100 mmol L^−1^ NaCl, 1 mmol L^−1^ DTT and 1% protease inhibitor cocktail), lysed by sonication on ice and then both proteins were purified using glutathione–sepharose resin (Pierce) as per manufacturer's instructions. The purified proteins were analyzed by SDS/PAGE.

*Mycobacterium smegmatis sigF*,* rsfA,* and *rsfB* were amplified using gene specific primers and cloned into pET28a at NcoI‐HindIII (*sigF*) and NdeI‐XhoI (*rsfA* and *rsfB*) enzyme sites. The clones were verified by restriction digestion and DNA sequencing. Recombinant pET28a carrying *sigF, rsfA, and rsfB* in fusion with N‐terminal His_6_ tag were transformed into *E. coli* pLysY^+^ cells separately and transformants were appropriately selected. Selected colonies of pET28a‐MS*sigF*, pET28a‐ MS*rsfA,* and pET28a‐MS*rsfB* were grown, proteins were overexpressed and cell lysates were prepared as described above.

Pull down experiments were performed using Pierce GST Protein Interaction Pull‐Down Kit (cat # PI21516) according to manufacturer's instructions. Purified GST‐MS*rsbW* and GST proteins (5 *μ*g each) were allowed to bind 50 *μ*L GST resins at 4°C for 1 h. GST proteins were used as negative control. After several washings (wash buffer 1) columns carrying GST‐MS*rsbW* and GST bound resins were incubated separately with total cell lysates containing overexpressed *M. smegmatis* SigF, RsfA, and RsfB in buffer (TBS: 50 mmol L^−1^ Tris pH 7.4, 100 mmol L^−1^ NaCl) at 4°C for 1 h with constant mixing. After washing five times with 400 *μ*L of wash buffer (wash buffer 1) the bound proteins were eluted in TBS containing 5 and 10 mmol L^−1^ reduced glutathione (RG). Eluted samples were boiled in 1X sample buffer, separated using 15% SDS‐PAGE and visualized by coomassie staining (Fig. 6). Individual bands were excised and analysed using MS/MS, which confirmed the identity of eluted proteins.

### Overexpression of **rsbW** from *Mycobacterium smegmatis* and *M. tuberculosis*

Anti‐sigma factors from *M. smegmatis* and *M. tuberculosis* were subcloned into *E. coli*/mycobacterial plasmid shuttle vector pMV261(Stover et al. 1991) to the downstream of *hsp60* promoter. *M. smegmatis* mc^2^155 wild type strain was subsequently transformed with pMV261‐MS*rsbW* and pMV261‐Mtb*rsbW* to generate MS:MS*rsbW* and MS:Mtb*rsbW* recombinant strains respectively. These strains were used for different analysis as described above.

### RNA isolation and labeling

*Mycobacterium smegmatis* strains were grown in Middlebrook 7H9 broth supplemented with 10% ADC, 0.2% glycerol and 0.05% Tween‐80 at 37°C. Aliquots were removed at exponential (~0.8 OD_600_) and stationary (~2.8 OD_600_) phase. Cells were harvested by centrifugation at 2500 × g for 5 min and RNA was extracted using Trizol (Invitrogen, USA), as described earlier (Singh and Singh 2009). The RNA was resuspended in 50 *μ*l of RNasefree water. RNA concentration and purity was determined using the NanoDrop^®^ ND‐1000 spectrophotometer (NanoDrop Technologies) and the integrity of total RNA was verified on an Agilent 2100 Bioanalyzer using the RNA 6000 Nano LabChip (Agilent Technologies). RNA was stored at −80°C until use. For labeling, RNA was polyadenylated using Poly (A) polymerase tailing kit (Cat # PAP5104H, Epicentre Biotechnologies) essentially as per manufacturer's instructions. Postpolyadenylation RNA was precipitated with ethanol, washed with 70% ethanol, dried at RT, and dissolved in nuclease free water. RNA concentration was estimated using NanoDrop and kept at −80°C until further use. Quick‐Amp Labeling kit (Agilent technologies) was used for cDNA synthesis and subsequent amplification and labeling by in vitro transcription was done as per one‐color labeling protocol (Agilent, version 5.5). Briefly, 0.5 *μ*g of each of the RNA sample was converted to double stranded cDNA using oligo dT primer with T7 polymerase promoter. RNA samples were mixed with T7 primers and final volume of each reaction was made up to 11.5 *μ*l with nuclease free water. Samples were denatured at 65°C for 10 min and placed on ice for 5 min. cDNA master mix was added to each sample and reactions were kept at 40°C for 2 h followed by incubation at 65°C for 15 min and on ice for 5 min. Then 60 *μ*l of transcription mix was added to each reaction and incubated at 40°C for 2 h. cRNA was generated by in vitro transcription using T7 RNA polymerase and the dye Cy3‐CTP was incorporated during this step. Labeled cRNA was purified using RNeasy Mini kit (Qiagen, India) and their quality was assessed for yields and specific activity using NanoDrop. Specific activity was calculated as picomole of dye/*μ*g of cRNA. Specific activity of ≥ 6.5 was considered optimal and used for hybridization.

### Microarray slides, hybridization, and scanning

Complete microarray experiment was carried out in technical collaboration with Genotypic Solution, Bangalore, India, official service partner of Agilent Technologies (USA). Array was spotted using 60 mer oligo probes (features) in 8 x15K format (Ref No: AMADID: 016421). Average number of probes per gene in each array is 3. Probes were designed in such a way that multiple probes for a given gene specifically hybridize to different parts of the transcript. Each array carried Agilent proprietary probes for quality control purpose. *M. smegmatis* microarray slides were hybridized with the labeled cRNA. Before hybridization 0.6 *μ*g of each Cy3 labeled cRNAs were fragmented to uniform size of 200 bp to avoid folding up of long transcripts and also remove any steric hindrance which may arise due to secondary structure in long RNA molecules during hybridization. Fragmentation and hybridization were carried out using the Gene Expression Hybridization kit (Part # 5188–5242, Agilent Technologies). Hybridization was carried out in Agilent's Surehyb Chambers at 65°C for 16 h. After hybridization slides were washed using Agilent Gene Expression wash buffers, first at RT and then twice at 37°C. Slides were quickly dried and scanned using the Agilent Microarray Scanner G Model G2565BA at 5 micron resolution. The images were manually verified and found to be devoid of uneven hybridization, streaks, blobs, and other artifacts.

### Feature extraction and data analysis

Data extraction from images was done using Feature Extraction software v 10.5.1.1 (Agilent). Feature extracted data were analyzed using GeneSpring GX v 7.3.1 software (Agilent). Normalization of the data was done in GeneSpring GX using the recommended one color Per Chip and Per Gene Data Transformation: Set measurements <0.01 to 0.01 per Chip: Normalize to 50th percentile per Gene: Normalize to Specific Samples. The gene expression ratio (Δ*sigF*/WT) of ≤ 0.5 or ≥2.0 (*P *≤* *0.05) was considered differentially regulated and filtered from the data. Fold‐chage refers to expression ratio of Δ*sigF* strain to wild‐type and is expressed in log2. Ratios were tested for significance using student *T*‐test from Agilent's Gene Spring GX version 7.3 software.

### Real‐time reverse transcription‐PCR (RT‐PCR) analyses

RNA was extracted from exponential and stationary phase cultures of *M. smegmatis* wild type and derivative strains (SFKO1, SFKO1/*sigF*, MS:MS*rsbW* and MS:Mtb*rsbW*) as described earlier (Singh and Singh 2009). DNase treatement was carried out to remove any DNA contamination, and post‐treatment RNA was reverse transcribed using random primers and Transcriptor reverse transcriptase (Roche). qRT‐PCR was performed in triplicates using SYBR Green master mix on a Roche 480 LightCycler, as described previously (Singh and Singh 2009). Expression of target genes was normalized with the *sigA* transcript level. RNA samples that had not been reverse transcribed were included as controls in all the experiments. The mean relative expression levels and SD were determined from the data generated from two different experiments. Each experiment was set up in triplicates.

### Microarray data accession number

All experimental details and data have been deposited at the Gene Expression Omnibus (GEO, NCBI) under accession number GSE19774.

### Statistical analysis

Significant differences between experimental groups were determined using Student's *t*‐test (GRAPHPAD PRISM 5, GraphPad Software, Inc., La Jolla, CA). For all analyses, a *P*‐value of <0.05 was considered statistically significant.

## Conflicts of Interest

The authors declare no conflict of interest.

## Supporting information


**Data S1.** Log phase and stationary base.

**Figure S1.** Schematic of *sigF* locus and construction of *sigF* mutant.

**Figure S2.** TLC profile of the de‐*O*‐acetylated GPLs, extracted from the *Mycobacterium smegmatis* WT (MS) and mutant strain (SFKO1), as described in methods. dGPL I, II, III, and IV, starting from the solvent front. Mass spectra profile of GPLs (I, II, III, and IV) extracted from *M. smegmatis* wild type (A) *and* Δ*sigF* mutant (B).

**Figure S3**. Real time RT‐PCR analysis of select genes from microarray data that were found to be down‐regulated in Δ*sigF* mutant.
