# Supplementary material for: Characterization of Mycobacterium smegmatis sigF mutant and its regulon: overexpression of SigF antagonist (MSMEG_1803) in M. smegmatis mimics sigF mutant phenotype, loss of pigmentation, and sensitivity to oxidative stress
Source: Microbiologyopen. 2015 Oct 5;4(6):896–916. doi: 10.1002/mbo3.288 (PMC4694148; doi:10.1002/mbo3.288)
Supplement: Supplementary file 2 — Figure S1. Schematic of sigF locus and construction of sigF mutant. [file MBO3-4-0896-s002.ppt]

## Slide 1
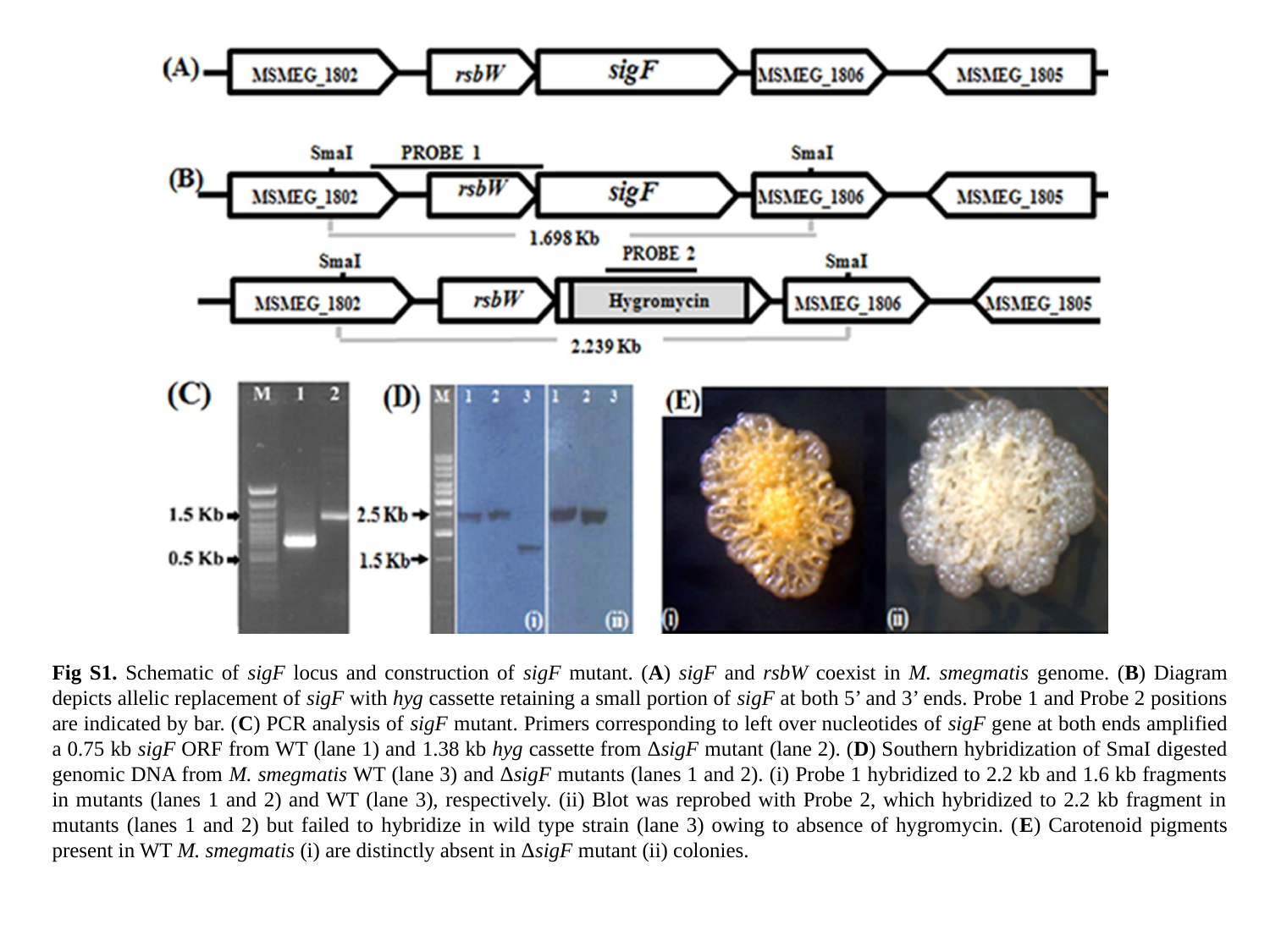

Fig S1. Schematic of sigF locus and construction of sigF mutant. (A) sigF and rsbW coexist in M. smegmatis genome. (B) Diagram depicts allelic replacement of sigF with hyg cassette retaining a small portion of sigF at both 5’ and 3’ ends. Probe 1 and Probe 2 positions are indicated by bar. (C) PCR analysis of sigF mutant. Primers corresponding to left over nucleotides of sigF gene at both ends amplified a 0.75 kb sigF ORF from WT (lane 1) and 1.38 kb hyg cassette from ΔsigF mutant (lane 2). (D) Southern hybridization of SmaI digested genomic DNA from M. smegmatis WT (lane 3) and ΔsigF mutants (lanes 1 and 2). (i) Probe 1 hybridized to 2.2 kb and 1.6 kb fragments in mutants (lanes 1 and 2) and WT (lane 3), respectively. (ii) Blot was reprobed with Probe 2, which hybridized to 2.2 kb fragment in mutants (lanes 1 and 2) but failed to hybridize in wild type strain (lane 3) owing to absence of hygromycin. (E) Carotenoid pigments present in WT M. smegmatis (i) are distinctly absent in ΔsigF mutant (ii) colonies.
