# Supplementary material for: Characterization of Mycobacterium smegmatis sigF mutant and its regulon: overexpression of SigF antagonist (MSMEG_1803) in M. smegmatis mimics sigF mutant phenotype, loss of pigmentation, and sensitivity to oxidative stress
Source: Microbiologyopen. 2015 Oct 5;4(6):896–916. doi: 10.1002/mbo3.288 (PMC4694148; doi:10.1002/mbo3.288)
Supplement: Supplementary file 3 — Figure S2. TLC profile of the de‐O‐acetylated GPLs, extracted from the Mycobacterium smegmatis WT (MS) and mutant strain (SFKO1), as described in methods. dGPL I, II, III, and IV, starting from the solvent front. Mass spectra profile of GPLs (I, II, III, and IV) extracted from M. smegmatis wild type (A) and ΔsigF mutant (B). [file MBO3-4-0896-s003.doc]

Fig. S2

**
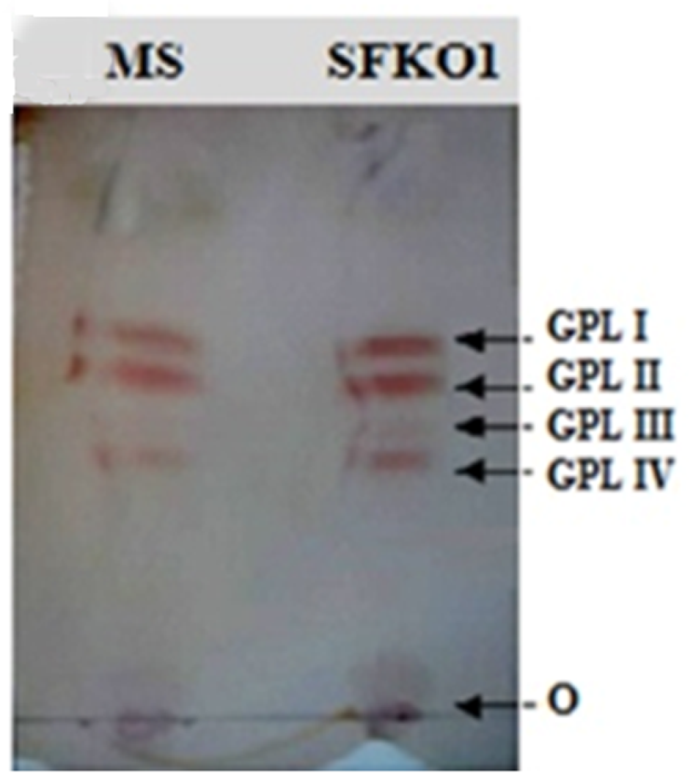
**

TLC profile of the de-*O*-acetylated GPLs, extracted from the *M. smegmatis* WT (MS) and mutant strain (SFKO1), as described in methods. dGPL I, II, III, and IV, starting from the solvent front.


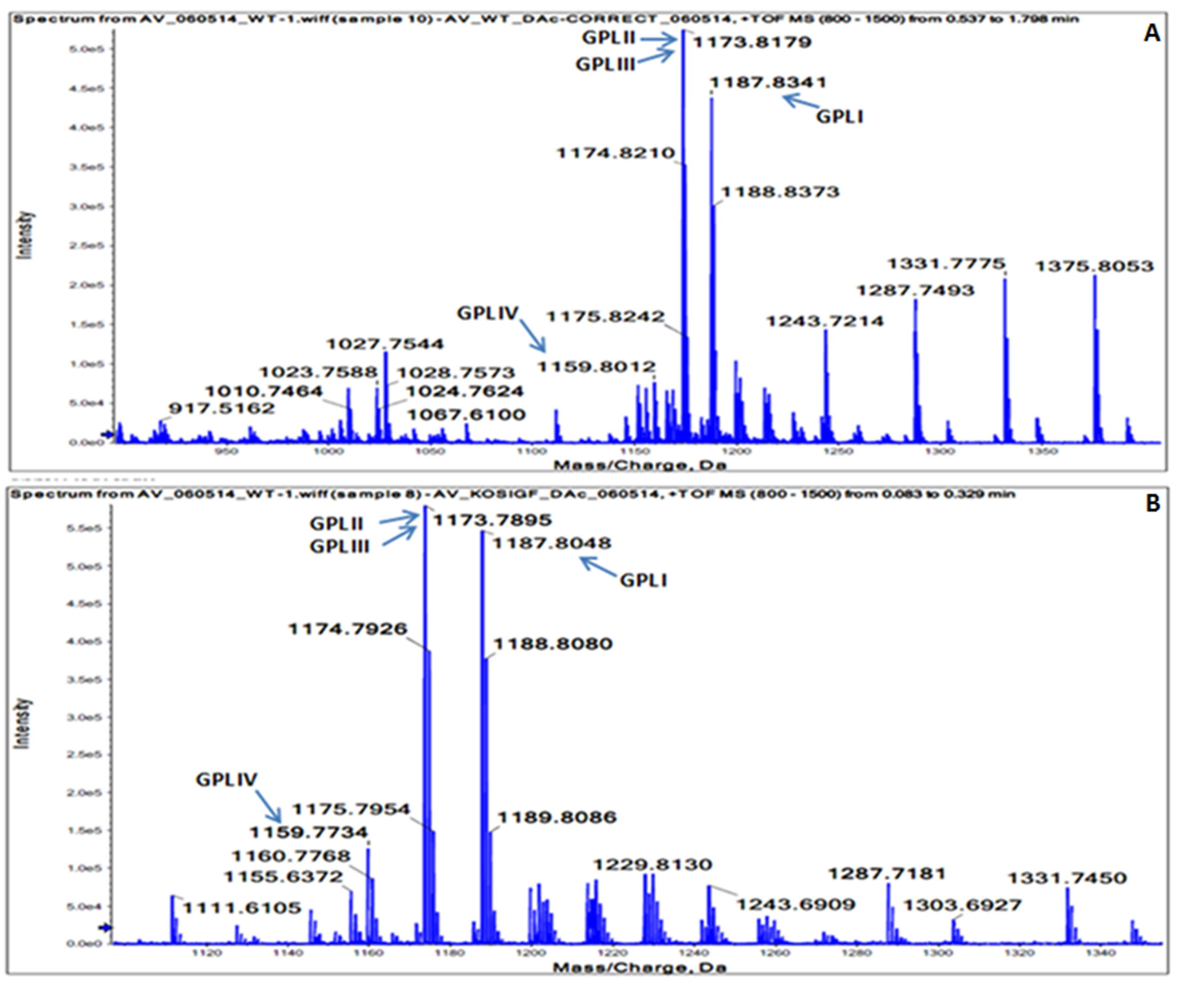


Mass spectra profile of GPLs (I, II, III, and IV) extracted from *M. smegmatis* wild type (A) *and* Δ*sigF* mutant (B).
