# Supplementary material for: Characterization of Mycobacterium smegmatis sigF mutant and its regulon: overexpression of SigF antagonist (MSMEG_1803) in M. smegmatis mimics sigF mutant phenotype, loss of pigmentation, and sensitivity to oxidative stress
Source: Microbiologyopen. 2015 Oct 5;4(6):896–916. doi: 10.1002/mbo3.288 (PMC4694148; doi:10.1002/mbo3.288)
Supplement: Supplementary file 4 — Figure S3. Real time RT‐PCR analysis of select genes from microarray data that were found to be down‐regulated in ΔsigF mutant. [file MBO3-4-0896-s004.ppt]

## Slide 1
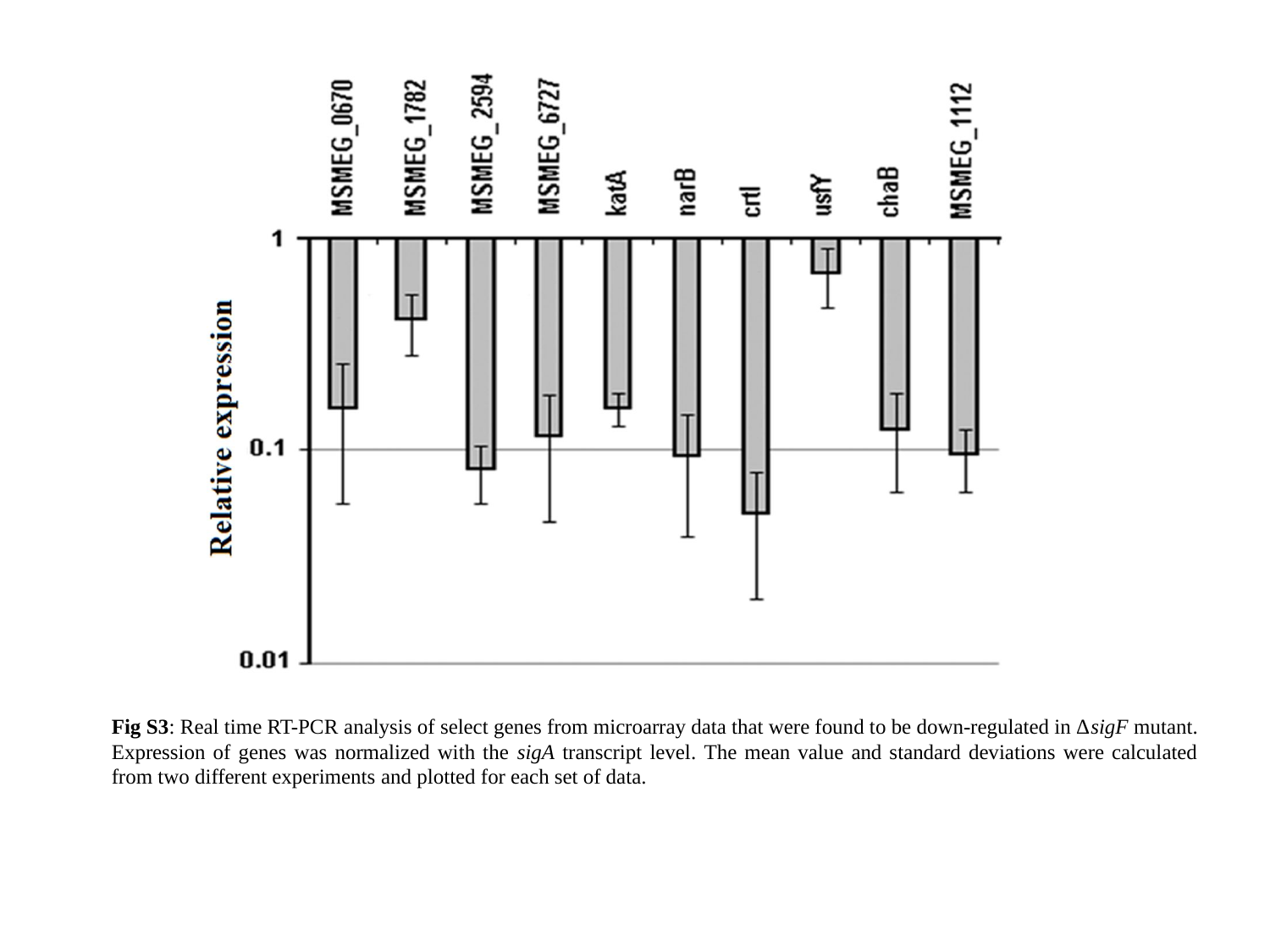

Fig S3: Real time RT-PCR analysis of select genes from microarray data that were found to be down-regulated in ΔsigF mutant. Expression of genes was normalized with the sigA transcript level. The mean value and standard deviations were calculated from two different experiments and plotted for each set of data.
